# Evaluation and Interpretation of Transcriptome Data Underlying Heterogeneous Chronic Obstructive Pulmonary Disease

**DOI:** 10.5808/GI.2019.17.1.e2

**Published:** 2019-03-31

**Authors:** Seokjin Ham, Yeon-Mok Oh, Tae-Young Roh

**Affiliations:** 1 Department of Life Sciences, POSTECH, Pohang 37674, Korea; 2 Department of Pulmonary and Critical Care Medicine, Asan Medical Center, University of Ulsan College of Medicine, Seoul 05505, Korea; 3 Division of Integrative Biosciences and Biotechnology, POSTECH, Pohang 37674, Korea

**Keywords:** chronic obstructive pulmonary disease, deconvolution, gene co-expression, gene heterogeneity, protein sublocalization

## Abstract

Chronic obstructive pulmonary disease (COPD) is a type of progressive lung disease, featured by airflow obstruction. Recently, a comprehensive analysis of the transcriptome in lung tissue of COPD patients was performed, but the heterogeneity of the sample was not seriously considered in characterizing the mechanistic dysregulation of COPD. Here, we established a new transcriptome analysis pipeline using a deconvolution process to reduce the heterogeneity and clearly identified that these transcriptome data originated from the mild or moderate stage of COPD patients. Differentially expressed or co-expressed genes in the protein interaction subnetworks were linked with mitochondrial dysfunction and the immune response, as expected. Computational protein localization prediction revealed that 19 proteins showing changes in subcellular localization were mostly related to mitochondria, suggesting that mislocalization of mitochondria-targeting proteins plays an important role in COPD pathology. Our extensive evaluation of COPD transcriptome data could provide guidelines for analyzing heterogeneous gene expression profiles and classifying potential candidate genes that are responsible for the pathogenesis of COPD.

## Introduction

COPD, or chronic obstructive pulmonary disease, is a type of obstructive lung disease characterized by long-term poor airflow [1]. It is a general term referring to chronic bronchitis, emphysema, and refractory (non-reversible) asthma. These progressive lung diseases are commonly characterized by increased shortness of breath, frequent coughing, increased breathlessness, and wheezing. COPD may be caused by a variety of environmental factors, such as air pollution, secondhand smoke, dust, fumes, and chemicals [2]. It is expected that diverse causes and symptoms of COPD may lead to heterogeneous gene expression profiles in individual COPD patients, as mentioned by Wedzicha [3].

Currently, more than 70% of COPD patients suffer from limited physical activity, and 50% among them can not lead a normal life [4,5]. In 2015, COPD ranked as the third leading cause of death worldwide, and it is expected that the mortality from COPD will increase greatly by 2030 [1].

Smoking causes about 80% to 90% of all deaths from COPD [2]. Chemical compounds in tobacco smoke may impair immunity to respiratory infections and increase the risk of lung damage. The number of female COPD cases is increasing due to the use of tobacco in some high-income countries and exposure to air pollution in low-income countries [5]. However, 25% of all COPD patients are never-smokers or passive smokers [5]. Genetic factors also contribute to the development of COPD. For example, alpha-1 antitrypsin, a serum serine protease inhibitor, functions to protect the lung from neutrophil elastase, and its deficiency allows chronic, uninhibited inflammation in the lung, leading to emphysema, along with chronic bronchitis [6].

The prevalence of COPD is well documented. The diagnostic assessment of COPD, as proposed by the Global Initiative for Chronic Obstructive Lung Disease (GOLD), is based on 4 multiple factors, such as the patient’s level of symptoms, the extent of airflow obstruction, spirometric abnormality, and the identification of comorbidities [1]. About 12 million adults in the United States are diagnosed with COPD, and 1% of them lose their life from it every year. Also, another 12 million people in the United States are regarded as having undiagnosed COPD [7]. However, most undiagnosed people are estimated to be in the mild or moderate stage of COPD and are not likely to be detected. Many case studies have considered alternative diagnostic aspects, which do not seem enough to cover the whole spectrum of COPD [2,8]. For example, inflammatory markers in COPD from the Bergen COPD cohort study have been used for the early diagnosis of COPD [9].

Recently, major biological and clinical discoveries have been allowed by great technical advances in next-generation sequencing techniques. Kim *et al*. [10] analyzed RNA sequencing (RNA-seq) data of 98 COPD lung tissue samples and 91 normal samples classified by the GOLD definition. In this study, they identified differentially expressed genes (DEGs) and isoforms (DEIs) between COPD and normal tissue. But, DEGs and DEIs could not be used for distinguishing COPD from normal tissue, probably due to the heterogeneity of the COPD samples.

Here, we established a new transcriptome analysis pipeline to remove heterogeneity and find suitable markers to clearly separate COPD from normal tissue. The removal of heterogeneity enabled us to detect emergent gene expression changes and protein interaction subnetworks that were missed in the previous study. Especially, the importance of mitochondrial proteins was revitalized through our analysis regarding co-expression relationships and changes in the subcellular localization of proteins. The analysis pipeline used in this study could be used to classify heterogeneous gene expression profiles and predict potential candidates for COPD pathogenesis.

## Methods

### Exploratory analysis of gene expression profiles

Raw RNA-seq data from 98 male COPD and 91 normal samples were downloaded from the Gene Expression Omnibus database (GSE57148, https://www.ncbi.nlm.nih.gov/geo/). The reads were aligned to the human genome (hg19) using tophat (v2.0.9) and bowtie2 (v2.1.0.0), along with—segment-length 50—segment-mismatches 1 [11,12]. The expression levels of individual transcripts by fragments per kilobase of exon per million fragments mapped (FPKM) were calculated by Cufflinks (v2.21) [13]. A total of 1,420 DEGs previously identified by Kim *et al*. [10] were used for comparison purposes. Principal component analysis (PCA) was performed with DEGs, and a three-dimensional plot was drawn in R. p-values in the bar graph were estimated by student’s t test. After 500 repeats with the e1071 library in R, the classification power of certain genes was examined by building a naive Bayes model with 10-times cross-validation. The performance of individual classification models, estimating sensitivity and specificity, was measured by computing area under the curve (AUC) values with the Receiver Operating characteristic Curve in R (ROCR) package.

### Measurement of VJ recombination events

Unmapped reads were collapsed, such that repeatedly appearing reads were regarded as a single read. The read count of each sequence was sorted in descending order, and the top 10,000 reads were selected from individual samples. The reads corresponding to V, D, J regions of the B cell receptor (BCR) and T cell receptor (TCR) loci were selected by an immunoglobulin variable domain sequence analysis tool, called IgBlast (http://www.ncbi.nlm.nih.gov/igblast/) [14]. Then, VJ recombination events were considered, using only in-frame sequence reads of 6 V, D, J regions. Alpha and beta diversity levels were calculated by vegan and the betapart library in R, respectively. A dot plot and a violin plot were visualized using R. p-values were calculated by permutation test with 1,000 permutations.

### Pipeline to remove heterogeneity

To remove transcriptome heterogeneity, DeMix [15], a statistical tool for deconvolving mixed transcriptomes, was used for 2,803 variable genes with a coefficient of variation of over 0.5 due to the high requirement of computer memory. In order to identify DEGs confidently, three different tests (t-test, Wilcoxon test, and median difference test) were performed with 1,000 permutations. Using POINTILLIST [16], the three p-values from each test were integrated into one. Genes with absolute fold-change over 1.25 and p-values less than 0.01 in COPD and normal subjects were regarded as DEGs. Functional and pathway enrichment assays of DEGs were carried out by DAVID [17]. Biological terms with p-values less than 0.01 were considered significant.

### COPD-related subnetworks

PhenomeExpress [18] was used to build vital subnetworks in COPD. Phenotypes relevant to COPD were used as seeds to construct the subnetworks. The seed phenotypes were HP:0002875 (exertional dyspnea), HP:0006510 (chronic obstructive pulmonary disease), MP:0001183 (overexpanded pulmonary alveoli), MP:0001951 (abnormal breathing pattern), MP:0010959 (abnormal oxidative phosphorylation), MP:0010956 (abnormal mitochondrial ATP synthesis-coupled electron transport), and MP:0002499 (chronic inflammation). Information on protein interactions was extracted from ConsensusPathDB [19]. Functional enrichment of subnetworks was examined by the Biological Networks Gene Ontology tool 7 (BiNGO), an open-source Cytoscape (v2.8.1) plugin to assess over-representation of gene ontology terms in networks [20]. Subnetworks enriched with specific functions were selected for further consideration.

### Pipeline to predict protein subcellular localization

Protein subcellular localization was examined and predicted using the analysis scheme suggested by Liu and Hu [21] and support vector machine (SVM). Information on protein interactions and subcellular localization was obtained from ConsensusPathDB [19] and the Human Protein Atlas [22], respectively. Gene expression profiles were converted into a matrix of maximal information coefficients (MICs), and the relationships between proteins were calculated using maximal information-based nonparametric exploration statistics [23]. Training and prediction with SVM were carried out with the e1071 library in R. The MICs for a protein pair were computed in individual COPD and normal samples, and the protein pairs with an absolute difference (delta MIC > 0.4) of 2 MICs were randomly defined as differentially co-expressed gene pairs (DCGPs).

## Results

### Evaluation of heterogeneity in COPD data

**Fig. 1.**
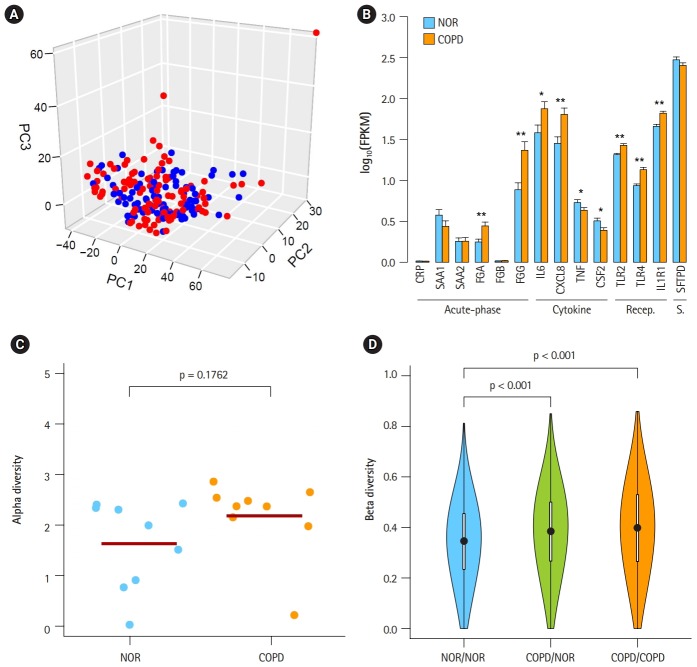
Heterogeneous chronic obstructive pulmonary disease (COPD) samples in the mild stage. (A) Principal component analysis plot depicting relative similarities between COPD samples (red) and normal (NOR) samples (blue) using previously identified differentially expressed genes. (B) Expression levels of COPD marker genes. Recep., receptor; S., surfactant; NOR. normal. *p < 0.01, **p < 0.0001 by student’s t-test. (C) Alpha diversity of VJ combinations in IGK. (D) Beta diversity showing an inverse relation with the compositional similarity between samples in terms of VJ combinations in IGK. p-values were calculated after 1,000 per bmutation.

In a previous study by Kim *et al*. [10], 1,420 DEGs between COPD and normal subjects were identified by student’s t-test and edgeR in Biocoductor [24]. To see the Euclidean distance and relatedness between COPD and normal subjects, PCA was performed (Fig. 1A). In a three-dimensional data space, it was hard to distinguish COPD samples from normal samples. Moreover, three principal components explained less than one-half of the variability between samples (PC1, 0.422; PC2, 0.069; and PC3, 0.047). These results revealed 8 heterogeneity in the COPD samples and indicated that simple conventional DEG comparison was not enough to classify the samples.

To identify the status of COPD samples, the average expression levels of known COPD marker genes were examined (Fig. 1B) [25-31]. Genes encoding acute phase proteins, such as fibrinogen α (FGA) and fibrinogen γ (FGG), were up-regulated in COPD. The expression levels of the immune cytokines interleukin 6 (IL6) and CXCL8 (IL8) were also increased. Genes of immune receptors associated with smoking were highly expressed in COPD than in normal samples. However, the extent of changes was generally less than 2-fold, suggesting that these COPD samples were in the mild stage of COPD. Unusually, the expression levels of tumor necrosis factor (TNF; TNF-α) and CSF2 (granulocyte-macrophage colony-stimulating factor [GM-CSF]) were lower in COPD, which was different from previous observations [27,28].

By analyzing RNA-seq data, it was possible to measure recombination events in BCR and TCR loci. VJ recombination occurs in the primary lymphoid organs and involves the joining of the variable (V) and joining (J) chains, resulting in the variation of amino acid sequences in the antigen-binding regions of BCRs and TCRs. By using IgBlast [14], frequent VJ recombination events in the immunoglobulin K (IGK) locus were identified. Alpha diversity represents how many components constitute a particular complex within a sample. In contrast, beta diversity is the compositional dissimilarity between samples. The alpha diversity levels of the IGK locus indicated that COPD samples contained marginally higher combinatorial diversity than normal samples (Fig. 1C). Besides, beta diversity levels showed lower similarity between COPD samples compared with normal samples (Fig. 1D). Normal samples were more similar to each other than to COPD samples. Other immunoglobulins (IGH and IGL) and TCRs (TCRA and TCRB) showed similar patterns in alpha and beta diversity levels as IGK (Supplementary Fig. 1 and 2). From these analyses, the COPD samples could be regarded 9 as heterogeneous in the mild stage of COPD.

### Reduction of heterogeneity of COPD samples

**Fig. 2.**
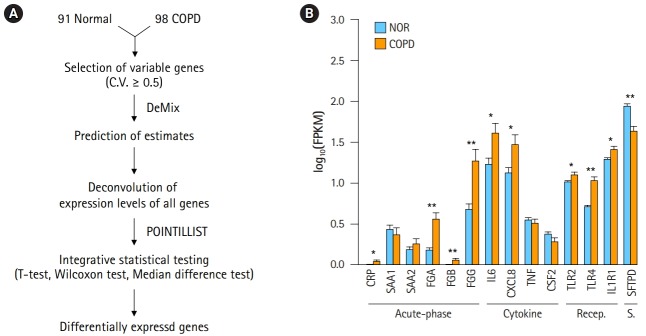
Deconvolution of chronic obstructive pulmonary disease (COPD) samples increases the difference between COPD and normal (NOR) tissue. (A) Deconvolution process to identify differentially expressed genes. (B) Expression levels of COPD marker genes after deconvolution. Recep., receptor; S., surfactant; NOR. normal. *p < 0.01, **p < 0.0001 by student’s t-test.

A workflow, including the prediction of estimates and the deconvolution process, was set up to resolve the issue of complexity (Fig. 2A). The critical steps in the pipeline were the prediction of estimates and deconvolution. The deconvolution process was originally designed to estimate the proportions of known sample types in a mixture of multiple samples. By assuming the RNA-seq data of our 98 COPD and 91 normal samples to be a mixture, the deconvolution process was applied to extract the unique profile of COPD samples. Having many samples was helpful to increase the accuracy. Then, integrative statistical test was performed to identify confident DEGs. p-values from the three tests were combined by POINTILLIST [16].

In order to confirm the effect of the deconvolution, expression levels of known marker genes were re-evaluated (Fig. 2B). The p-values of gene expression differences in the FGA, FGG, IL6, and CXCL8 genes were not changed. Immune receptors associated with smoking kept their higher expression levels in COPD versus normal tissue. However, in contrast to Fig. 1B, C-reactive protein (CRP) and β-fibrinogen (FGB) were now up-regulated in COPD. SFTPD was significantly down-regulated in COPD. The expression levels of TNF and CSF2 were not significantly different between COPD and normal tissue. These results suggest that the gene expression profiles of heterogeneous samples can be normalized in good agreement with known patterns through the deconvolution process.

### DEGs and biologically relevant subnetworks

**Fig. 3.**
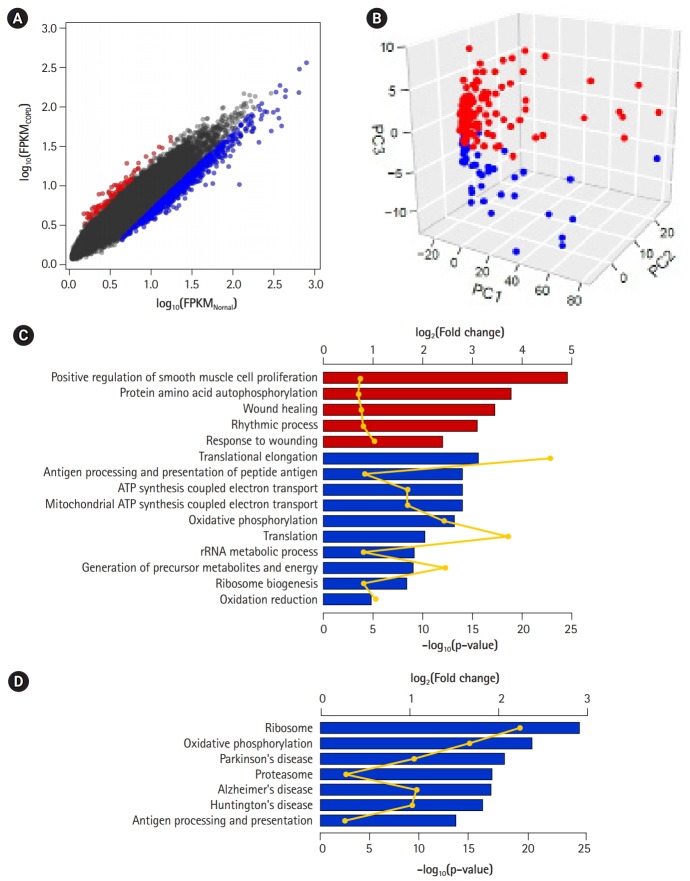
Differentially expressed genes (DEGs) between chronic obstructive pulmonary disease (COPD) and normal tissue. (A) Scatterplot of gene expression levels. Red and blue dots represent up-regulated and down-regulated genes in COPD compared with normal tissue, respectively. (B) Principal component analysis plot depicting relative similarities between COPD samples (red) and normal samples (blue) using DEGs. (C, D) Biological functions (C) and pathways (D) highly enriched in up-regulated (red) and down-regulated (blue) genes. Individual bars demonstrate fold-changes relative to background, and lines display their p-values.

DEGs—80 up-regulated genes and 757 down-regulated genes in COPD—could be 10 identified by applying the following conditions: genes with absolute fold-change over 1.25 and p-values less than 0.01 (Fig. 3A, Supplementary Table 1). Of them, 66 (82.5%) up-regulated genes and 501 (66.2%) down-regulated genes overlapped with the 1,420 genes previously identified by Kim *et al*. [10]. However, PCA of the DEGs showed a clear difference between COPD samples and normal samples (Fig. 3B). These DEGs might explain the variability between samples better than the previously identified DEGs (PC1, 0.702; PC2, 0.031; and PC3, 0.018). Accordingly, the performance of the prediction model with DEGs (AUC) increased from 0.793 to 0.931.

To detect biological functions or pathways closely related to specific genes, we performed enrichment assays with DEGs (Fig. 3C and 3D). A relatively small number of up-regulated genes in COPD were related to several functions, such as smooth muscle cell proliferation, protein autophosphorylation, and wound healing, as previously shown. On the other hand, down-regulated genes were associated with translational elongation, antigen processing and presentation, and oxidative phosphorylation coupled with electron transport in mitochondria. Additionally, in terms of biological pathways, down-regulated genes were linked to the ribosome, oxidative phosphorylation, the proteasome, and a couple of neurodegenerative disorders.

**Fig. 4.**
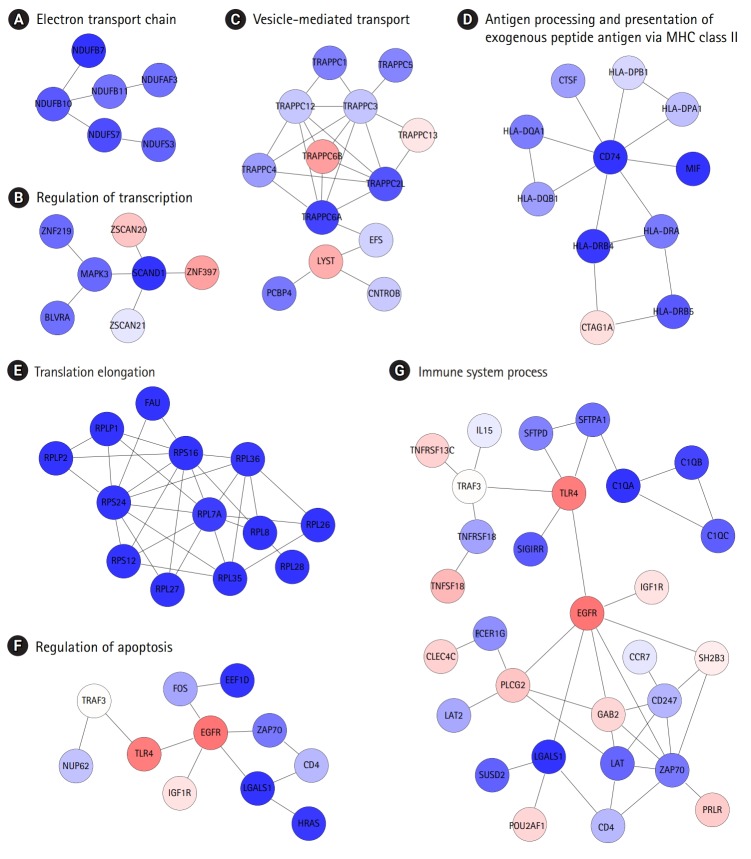
Biologically relevant subnetworks in consideration of certain disease phenotypes and gene expression changes. (A–G) Letters at the top are the most highly enriched biological functions in individual subnetworks.

The identification of protein interaction subnetworks using the transcriptome can provide useful information on interaction modules for specific functions. Reliable subnetworks were constructed by PhenomeExpress in combination with gene expression profiles and disease-related phenotypes [18]. There were five meaningful subnetworks significantly enriched with specific functions (Fig. 4A-4D). The largest subnetwork was too complex to interpret (Supplementary Fig. 3), and it was further divided into three subnetworks (Fig. 4E-4G). Functions, such as electron transport chain and translation elongation, were detected in 11 subnetworks, as with DEGs. In contrast, functions related to the regulation of transcription, vesicle-mediated transport, regulation of apoptosis, and immune system processes, were only observed in subnetworks. Whereas the term ‘general antigen presenting and presentation’ was enriched in DEGs, their function was confined to MHC class II in a subnetwork.

### DCGPs and protein sublocalization changes

**Fig. 5.**
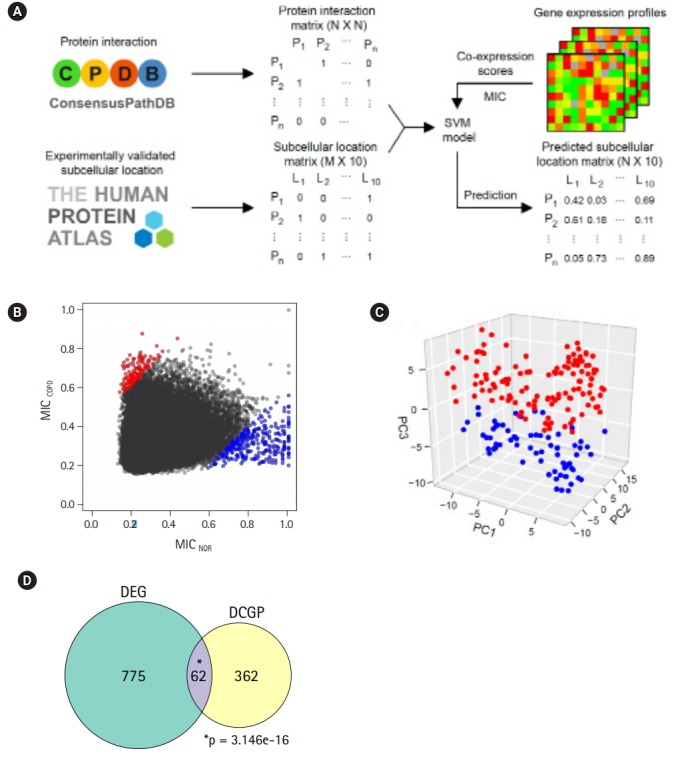
Differentially expressed pairs (DEPs) between chronic obstructive pulmonary disease (COPD) and normal (NOR) samples. (A) Pipeline to predict protein sublocalization from gene expression profiles. (B) Maximal information coefficient (MIC) scores for describing coexpression changes between two genes. Red and blue dots represent up-regulated and downregulated pairs in COPD compared with NOR samples, respectively. (C) Principal component analysis plot depicting relative similarities between COPD (red) and NOR samples (blue) using DEPs. (D) Venn diagram showing overlap between differentially expressed genes (DEGs) and DEPs. DCGP, differentially co-expressed gene pairs. p-values were calculated by hypergeometric test.

One emergent subnetwork associated with vesicle-mediated transport might be linked to the possibility that changes in protein subcellular localization play an important role in COPD development. Protein localization was predicted, based on gene expression profiles (Fig. 5A). To construct a co-expression network, gene expression profiles were converted into another format, and co-expression relationships of gene pairs were measured using MICs [23].

Two genes were regarded as DCGPs if the absolute MIC changes between COPD and normal subjects was greater than 0.4. Under this condition, 139 up-regulated pairs and 303 down-regulated pairs in COPD could be identified (Fig. 5B, Supplementary Table 2). In PCA with the DCGPs, there was a clear difference between COPD samples and normal samples (Fig. 5C), even though they showed variability between samples that was not large as with the DEGs (PC1, 0.102; PC2, 0.008; and PC3, 0.005). The prediction model with the DCGPs exhibited good performance (AUC, 0.946). Sixty-two genes among 424 genes in 442 DCGPs overlapped significantly with DEGs (hypergeometric test, p= 3.146e-16), but the remaining 362 genes were not matched to DEGs (Fig. 5D). These results imply that DCGPs could be complementary to DEGs for understanding gene expression profiles.

**Fig. 6.**
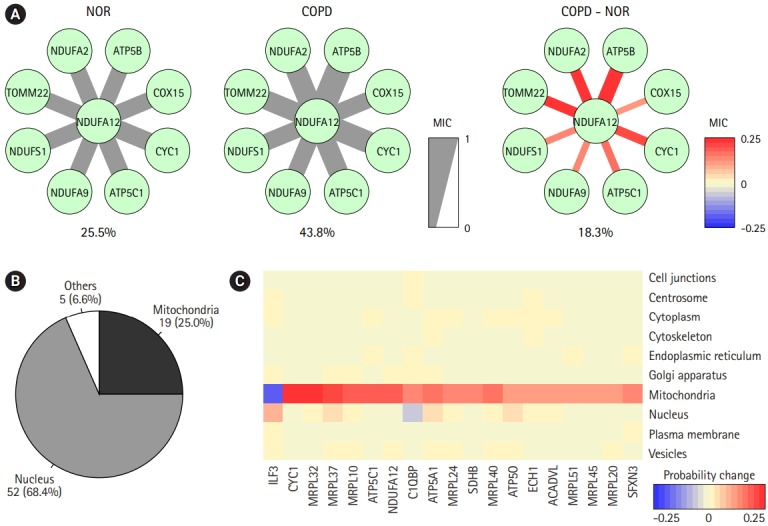
Prediction of protein subcellular localization changes between chronic obstructive pulmonary disease (COPD) and normal (NOR) samples. (A) The correlation scores between NDUFA12 and other mitochondrial proteins in NOR and COPD samples. The thickness and color of the edges were determined by maximal information coefficient (MIC). (B) Genes with significant subcellular localization changes between COPD and NOR samples. (C) Heatmap demonstrating probability of changes in mitochondria-related proteins.

The prediction of protein subcellular localization is exemplified in Fig. 6A. A mitochondrial protein, NDUFA12, was selected, because it had 8 interacting proteins and showed coherent changes in all interactions. The predicted chance of NDUFA12 translocating toward 12 mitochondria was increased in COPD (43.8%), compared with normal tissue (25.5%). In COPD, protein interactions between NDUFA12 and other mitochondrial proteins were reinforced. However, the actual protein sublocalization changes were expected to be much more complex when considering all protein interactions. Our analysis workflow was designed to include all protein interactions and thus predicted the probabilities of 10 subcellular locations of each protein for each status (Fig. 5A). Out of 76 significant subcellular localization changes between COPD and normal tissue, 19 (25.0%) were related to mitochondria and 52 (68.4%) were related to the nucleus (Fig. 6B).

The predicted probabilities of subcellular locations of the mitochondria-related proteins were examined (Fig. 6C). Except for ILF3, all proteins showed higher chances of localizing to mitochondria in COPD than in normal tissue. Seven of them were mitochondrial ribosomal proteins, and other proteins, such as CYC1, ATP5C1, NDUFA12, C1QBP, ATP5A1, SDHB, ATP5O, ECH1, ACADVL, and SFXN3, acted on the matrix of mitochondria. Collectively, proteins targeting mitochondria might be influenced by mitochondrial dysfunction in COPD.

## Discussion

COPD is a complex and heterogeneous disease, and thus, it is not easy to investigate the pathogenesis and diagnosis of COPD [3]. Previously, Kim *et al*. [10] performed RNA-seq analysis with 98 COPD samples and 91 normal samples. However, DEGs identified by a simple calculation of fold-change in gene expression level might not be useful—especially in this study: COPD versus normal (Fig. 1A). The heterogeneity might be attributed to a number of different pathological processes. Among them, bacteria have been reported as one of the major causes in the exacerbation of COPD, contributing to the severe inflammatory response in the 13 airways [32]. While mild-to-moderate COPD exhibits higher diversity in the bacterial population [33], severe COPD shows lower diversity [34].

We confirmed that our COPD data were in the mild stage, based on the expression levels of known marker genes (Fig. 1B). These COPD samples exhibited a marginal but consistent rise in combinatorial diversity in all BCR and TCR loci (Fig. 1C, Supplementary Fig. 1). It is possible that increased diversity of the microbiome in the mild stage of COPD led to increased diversity of BCR and TCR loci. Accordingly, a lower level of similarity was observed between COPD samples compared with normal samples (Fig. 1D, Supplementary Fig. 2).

Our analysis pipeline to identify DEGs was performed considering two aspects: heterogeneity and confidence (Fig. 2A). First, computational deconvolution reduced the heterogeneity between COPD samples. Second, integrative statistical tests were applied to identify confident DEGs. It is known that a combination of t-test, Wilcoxon test, and median difference test can reduce the overestimation by removing biases [35], because conventional t-test-based tools tend to calculate p-values too optimistically when they are applied to a large number of samples [10].

By reducing the heterogeneity, gene expression profiles of COPD samples could become consistent with known expression patterns of marker genes. The DEGs that were identified in our pipeline were better in distinguishing COPD from normal subjects than previously defined DEGs (Fig. 3A and 3B). Through deconvolution, gene expression profiles among COPD samples could become consistent when examined, based on known expression patterns of marker genes. Acute-phase proteins, such as CRP, acute-phase serum amyloid A, and fibrinogens, are well known and are induced in response to inflammation and in COPD [26]. IL6, CXCL8 (IL8), TNF (TNF α), and CSF2 (GM-CSF) are airway inflammatory cytokines that are up-regulated in COPD patients [27,28]. The levels of immune receptors, such as TLR2, 14 TLR4, and IL1R1, increase with smoking [29], but the levels of surfactant protein D decrease [30]. Regardless of deconvolution, some acute-phase proteins (FGA, FGG), cytokines (IL6, CXCL8), and immune receptors were consistently up-regulated in COPD. However, CRP and FGB were up-regulated and SFTPD was down-regulated in COPD only after deconvolution. Moreover, TNF and CSF2 were no longer significantly down-regulated. These observations could be identified, because sample heterogeneity was considered in the analysis.

Up-regulated genes in COPD were related with to functions, such as smooth muscle cell proliferation, protein autophosphorylation, and wound healing (Fig. 3C), consistent with a previous report that oxidative stress-induced mitochondrial dysfunction induces inflammation and airway smooth muscle remodeling in COPD [36]. On the other hand, down-regulated genes in COPD were relevant to translational elongation, oxidative phosphorylation coupled to electron transport in mitochondria, and, in particular, neurodegenerative disorders (Fig. 3D), agreeing that COPD patients are likely to develop specific cognitive impairments [37].

Identification of protein interaction subnetworks shed light on specific functions of interaction modules related to the typical phenotypes of COPD (Fig. 4). Functions related to the regulation of transcription, vesicle-mediated transport, regulation of apoptosis, and immune system processes were only observed in subnetworks, not in DEGs. Furthermore, antigen presentation was more confined to MHC class II. In this analysis, the levels of MHC class II genes and some immune components decreased, whereas other immune components were down-regulated in COPD, showing the complexity of immune responses in COPD.

One attractive subnetwork associated with vesicle-mediated transport raised the question of whether protein subcellular localization plays some role in COPD. A group of proteins with subcellular localization changes in COPD were predicted by measuring co-expression scores using information on protein interaction and subcellular localization (Figs. 5 and 6). Interestingly, one-quarter of predicted changes were related to mitochondria, suggesting that proteins targeting mitochondria might be influenced by mitochondrial dysfunction. Mitochondrial ribosomal proteins and other proteins on the mitochondrial matrix were enriched in mitochondria in COPD cases.

Here, we used public gene expression profiles generated from COPD and normal subjects and re-evaluated the differential transcriptomes by removing sample heterogeneity. The overall data analysis revealed a group of gene expression changes that were missed in previous research. Co-expression relationships between conditions could be inferred from gene expression profiles and might be useful in classifying samples and predicting protein subcellular localization. In conclusion, COPD is a complex and heterogeneous disease. The newly identified DEGs in this study and DCGPs could partially explain COPD pathogenesis in the mild stage. We expect that our strategy of analyzing heterogeneous samples will be applicable to other systems.

## Supplementary Materials

Supplementary Fig. 1. Alpha diversity of VJ combinations in IGL (A), IGH (B), TCRA (C), and TCR (D). Chronic obstructive pulmonary disease (COPD) samples exhibit slightly increased combinatorial diversity compared with normal samples. p-values were calculated after 1,000 permutations.

Supplementary Fig. 2. Beta diversity of VJ combinations in IGL (A), IGH (B), TCRA (C), and TCR (D). Lower level of similarity is observed between chronic obstructive pulmonary disease (COPD) samples compared with normal samples. Normal samples are more similar to each other than to COPD samples. p-values were calculated after 1,000 permutations.

Supplementary Fig. 3. The largest biologically relevant subnetworks determined by certain disease phenotypes and gene expression changes, including subnetworks related to biological functions, such as translational elongation, regulation of apoptosis, and immune system processes.

Supplementary Table 1. List of differentially expressed genes after deconvolution

Supplementary Table 2. List of differentially expressed pairs determined by maximal information coefficient
