# Supplementary material for: Evaluation and Interpretation of Transcriptome Data Underlying Heterogeneous Chronic Obstructive Pulmonary Disease
Source: Genomics Inform. 2019 Mar 31;17(1):e2. doi: 10.5808/GI.2019.17.1.e2 (PMC6459164; doi:10.5808/GI.2019.17.1.e2)
Supplement: Supplementary Fig. 1. — Alpha diversity of VJ combinations in IGL (A), IGH (B), TCRA (C), and TCR (D). Chronic obstructive pulmonary disease (COPD) samples exhibit slightly increased combinatorial diversity compared with normal samples. p-values were calculated after 1,000 permutations. [file gi-2019-17-1-e2-suppl1.pdf]

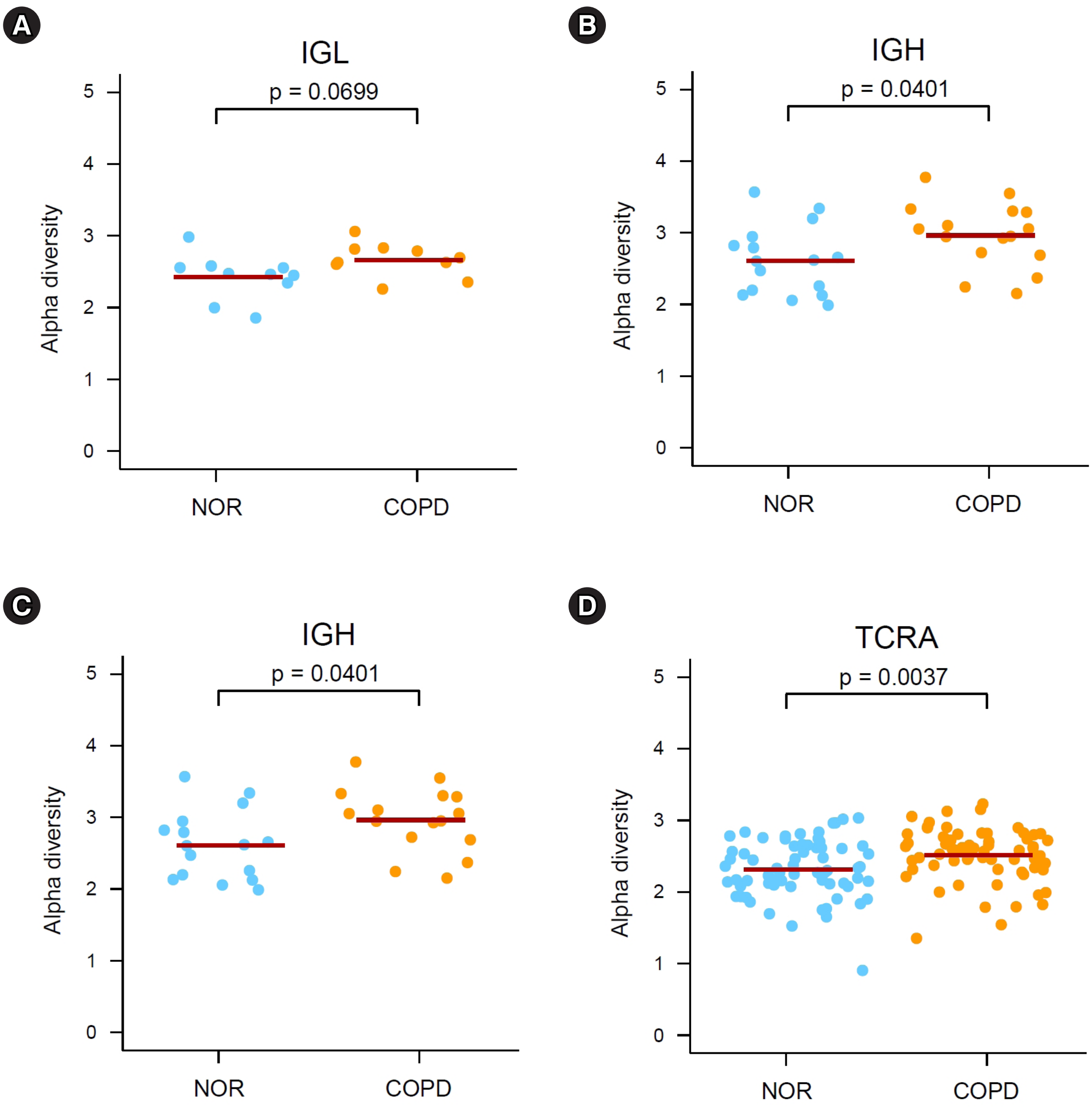

Supplementary Fig. 1. Alpha diversity of VJ combinations in IGL (A), IGH (B), TCRA (C), and TCR (D). Chronic obstructive pulmonary disease (COPD) samples exhibit slightly increased combinatorial diversity compared with normal (NOR) samples. p-values were calculated after 1,000 permutations.

https://doi.org/10.5808/GI.2019.17.1.e2
