# Supplementary material for: Evaluation and Interpretation of Transcriptome Data Underlying Heterogeneous Chronic Obstructive Pulmonary Disease
Source: Genomics Inform. 2019 Mar 31;17(1):e2. doi: 10.5808/GI.2019.17.1.e2 (PMC6459164; doi:10.5808/GI.2019.17.1.e2)
Supplement: Supplementary Fig. 2. — Beta diversity of VJ combinations in IGL (A), IGH (B), TCRA (C), and TCR (D). Lower level of similarity is observed between chronic obstructive pulmonary disease (COPD) samples compared with normal samples. Normal samples are more similar to each other than to COPD samples. p-values were calculated after 1,000 permutations. [file gi-2019-17-1-e2-suppl2.pdf]

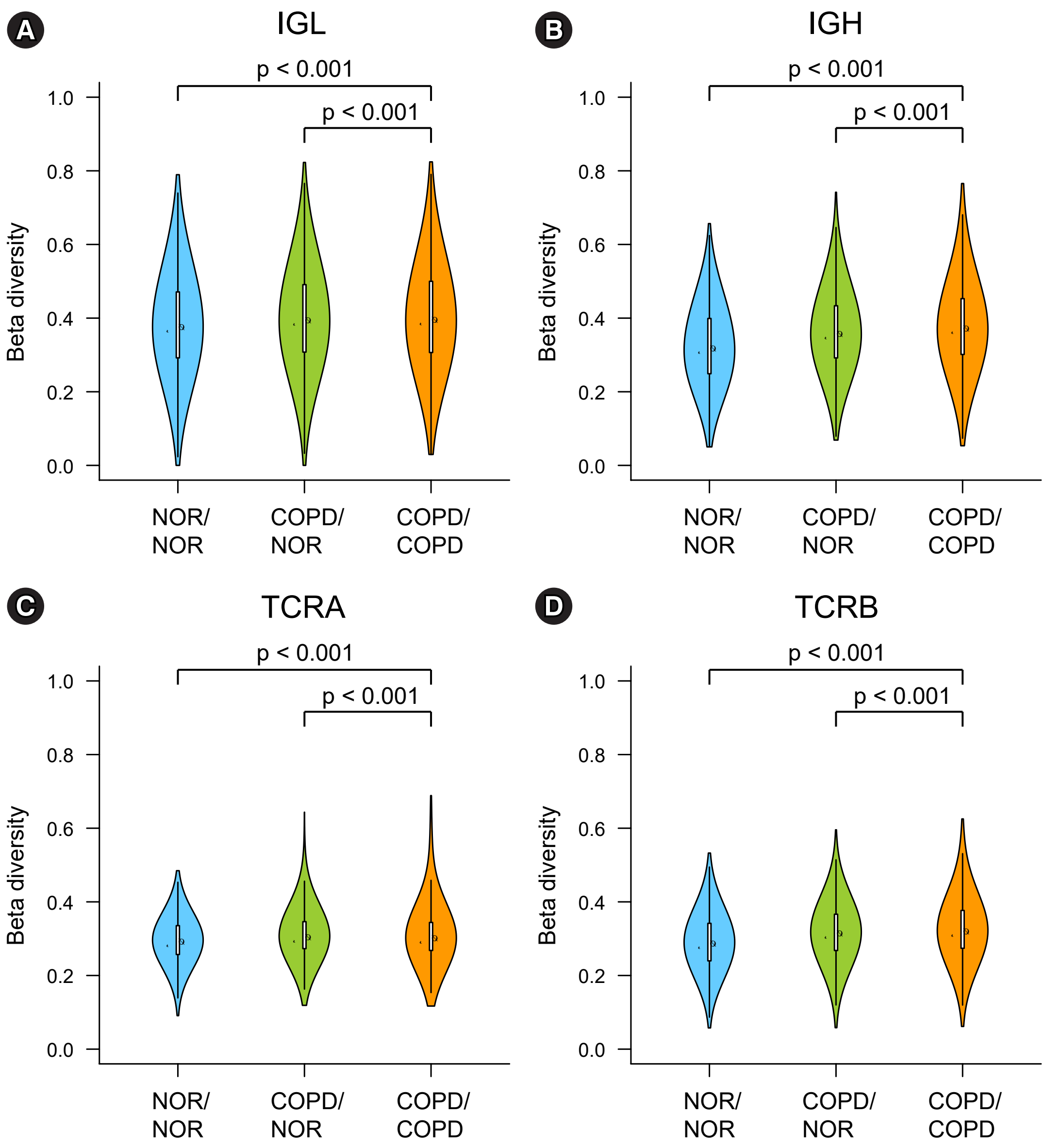

Supplementary Fig. 2. Beta diversity of VJ combinations in IGL (A), IGH (B), TCRA (C), and TCR (D). Lower level of similarity is observed between chronic obstructive pulmonary disease (COPD) samples compared with normal (NOR) samples. NOR samples are more similar to each other than to COPD samples. p-values were calculated after 1,000 permutations.

https://doi.org/10.5808/GI.2019.17.1.e2
