# Supplementary material for: Evaluation and Interpretation of Transcriptome Data Underlying Heterogeneous Chronic Obstructive Pulmonary Disease
Source: Genomics Inform. 2019 Mar 31;17(1):e2. doi: 10.5808/GI.2019.17.1.e2 (PMC6459164; doi:10.5808/GI.2019.17.1.e2)
Supplement: Supplementary Fig. 3. — The largest biologically relevant subnetworks determined by certain disease phenotypes and gene expression changes, including subnetworks related to biological functions, such as translational elongation, regulation of apoptosis, and immune system processes. [file gi-2019-17-1-e2-suppl3.pdf]

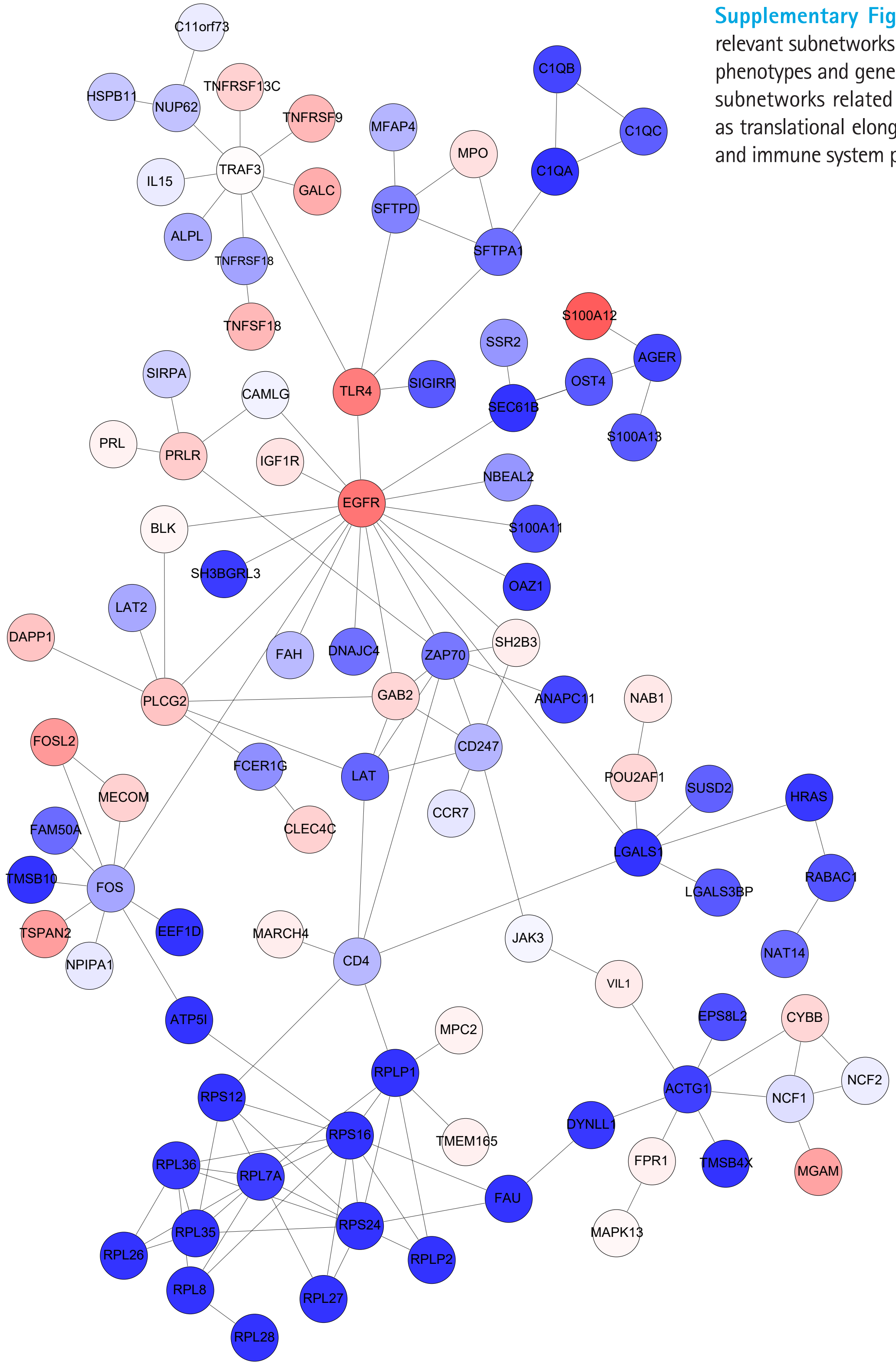

Supplementary Fig. 3. The largest biologically relevant subnetworks determined by certain disease phenotypes and gene expression changes, including subnetworks related to biological functions, such as translational elongation, regulation of apoptosis, and immune system processes.

https://doi.org/10.5808/GI.2019.17.1.e2
