# Supplementary material for: Evaluation and Interpretation of Transcriptome Data Underlying Heterogeneous Chronic Obstructive Pulmonary Disease
Source: Genomics Inform. 2019 Mar 31;17(1):e2. doi: 10.5808/GI.2019.17.1.e2 (PMC6459164; doi:10.5808/GI.2019.17.1.e2)
Supplement: Supplementary Table 1. — List of differentially expressed genes after deconvolution [file gi-2019-17-1-e2-suppl4.pdf]

Supplementary Table 1. List of differentially expressed genes after deconvolution

Gene	log10(Adjus	log10(Adjus	Fold chang	Combined p-value
AATK	1.025189	0.67593	-0.34926	5.44E-07
ABTB1	1.246771	0.878688	-0.36808	5.44E-07
ACAP3	0.962667	0.628372	-0.33429	5.44E-07
ACP5	1.798233	1.398043	-0.40019	5.44E-07
ACTA2	1.644757	1.27522	-0.36954	5.44E-07
ACTB	2.674842	2.213295	-0.46155	5.44E-07
ACTG1	2.40878	1.940589	-0.46819	5.44E-07
ACTG2	1.053504	0.728436	-0.32507	5.44E-07
ADIRF	1.886374	1.51171	-0.37466	5.44E-07
ADRM1	1.314155	0.955009	-0.35915	5.44E-07
AES	1.878266	1.502989	-0.37528	5.44E-07
AGAP3	1.349862	0.987894	-0.36197	5.44E-07
AGER	2.248495	1.789578	-0.45892	5.44E-07
AGPAT2	1.432603	1.081641	-0.35096	5.44E-07
AGRN	1.45292	1.122483	-0.33044	5.44E-07
AGTRAP	1.21336	0.832988	-0.38037	5.44E-07
AHRR	1.293302	0.912241	-0.38106	5.44E-07
AK1	1.261802	0.923499	-0.3383	5.44E-07
ALDH16A1	1.00369	0.615432	-0.38826	5.44E-07
ALDOA	1.813553	1.408374	-0.40518	5.44E-07
ALKBH7	1.106729	0.679356	-0.42737	5.44E-07
ALS2CL	1.136595	0.802961	-0.33363	5.44E-07
ANAPC11	1.29217	0.832797	-0.45937	5.44E-07
ANKRD13	0.998196	0.6615	-0.3367	5.44E-07
APOC1	1.874408	1.339411	-0.535	5.44E-07
APOE	2.011775	1.562746	-0.44903	5.44E-07
APOLD1	0.715199	1.052482	0.337283	1.21E-05
APRT	1.31802	0.813547	-0.50447	5.44E-07
ARF5	1.524256	1.047593	-0.47666	5.44E-07
ARFGAP1	1.261759	0.885042	-0.37672	5.44E-07
ARHGAP4	1.100262	0.746083	-0.35418	5.44E-07
ARHGDIA	1.72981	1.365264	-0.36455	5.44E-07
ARHGEF1	1.423074	1.027049	-0.39603	5.44E-07
ARL2	1.455761	1.095063	-0.3607	5.44E-07
ARL5B	0.481319	0.956074	0.474755	1.21E-05
ARL6IP4	1.446668	0.984932	-0.46174	5.44E-07
ARPC1B	1.746557	1.365892	-0.38066	5.44E-07
ARRDC1	1.244966	0.863305	-0.38166	5.44E-07
ARVCF	0.721603	0.383446	-0.33816	5.44E-07
ASPSCR1	0.996615	0.59934	-0.39728	5.44E-07
ASXL2	0.491946	0.880515	0.388569	1.21E-05
ATF4	1.786364	1.417322	-0.36904	5.44E-07
ATHL1	1.241405	0.766706	-0.4747	5.44E-07
ATOX1	1.541497	1.131509	-0.40999	5.44E-07
ATP5D	1.044489	0.616955	-0.42753	5.44E-07
ATP5G1	1.244824	0.919918	-0.32491	5.44E-07
ATP5G2	1.45232	1.043308	-0.40901	5.44E-07
ATP5I	1.90597	1.372369	-0.5336	5.44E-07
ATP5J	1.329185	0.984063	-0.34512	5.44E-07
ATP5J2	1.503528	1.094774	-0.40875	5.44E-07
ATP6V0B	1.476717	1.093241	-0.38348	5.44E-07
ATP6V0E1	1.900333	1.543516	-0.35682	5.44E-07
ATP6V1F	1.68005	1.229399	-0.45065	5.44E-07

https://doi.org/10.5808/GI.2019.17.1.e2

ATPIF1	1.787123	1.35002	-0.4371	5.44E-07
AUP1	1.453357	1.108765	-0.34459	5.44E-07
AURKAIP1	1.322177	0.816829	-0.50535	5.44E-07
B2M	3.290891	2.81391	-0.47698	5.44E-07
B3GAT3	0.698905	0.356532	-0.34237	5.44E-07
BAD	1.259242	0.809449	-0.44979	5.44E-07
BAX	1.350624	1.003032	-0.34759	5.44E-07
BCAM	1.620312	1.238408	-0.3819	5.44E-07
BCL7C	0.878573	0.509077	-0.3695	5.44E-07
BLVRA	1.459248	1.108979	-0.35027	5.44E-07
BLVRB	1.471844	1.042089	-0.42975	5.44E-07
BOLA3	1.052726	0.694619	-0.35811	5.44E-07
BRAT1	0.883134	0.515145	-0.36799	5.44E-07
BRI3	1.530004	1.179313	-0.35069	5.44E-07
BSG	1.824751	1.391891	-0.43286	5.44E-07
BST2	1.756083	1.294972	-0.46111	5.44E-07
C11orf68	1.02833	0.680036	-0.34829	5.44E-07
C12orf57	1.594911	1.253263	-0.34165	5.44E-07
C16orf13	1.099544	0.675141	-0.4244	5.44E-07
C19orf24	1.043516	0.71353	-0.32999	5.44E-07
C19orf33	1.67258	1.229102	-0.44348	5.44E-07
C19orf43	1.581974	1.157325	-0.42465	5.44E-07
C19orf53	1.325612	0.974747	-0.35086	5.44E-07
C19orf60	1.196735	0.705124	-0.49161	5.44E-07
C19orf70	0.939434	0.503572	-0.43586	5.44E-07
C1orf122	1.091437	0.711696	-0.37974	5.44E-07
C1QA	2.075674	1.542707	-0.53297	5.44E-07
C1QB	2.048	1.592525	-0.45547	5.44E-07
C1QC	1.934365	1.540512	-0.39385	5.44E-07
C1QTNF5	1.213278	0.835813	-0.37746	5.44E-07
C21orf33	1.322748	0.981323	-0.34143	5.44E-07
C2orf54	0.870391	0.494083	-0.37631	5.44E-07
C6orf1	1.055359	0.657179	-0.39818	5.44E-07
C6orf226	0.905211	0.483945	-0.42127	5.44E-07
C7orf50	1.232002	0.838345	-0.39366	5.44E-07
C8orf82	0.944386	0.594209	-0.35018	5.44E-07
C9orf142	1.023591	0.531874	-0.49172	5.44E-07
C9orf16	1.202716	0.73692	-0.4658	5.44E-07
CAMTA1	1.317139	0.974127	-0.34301	5.44E-07
CAPG	1.671924	1.321401	-0.35052	5.44E-07
CAPN15	0.94106	0.611141	-0.32992	5.44E-07
CAPNS1	1.736063	1.400964	-0.3351	5.44E-07
CAPS	0.986914	0.63408	-0.35283	5.44E-07
CARNMT1	0.421772	0.773826	0.352054	1.21E-05
CBR1	1.281867	0.928019	-0.35385	5.44E-07
CCDC106	0.641012	0.315254	-0.32576	5.44E-07
CCDC12	1.290033	0.866402	-0.42363	5.44E-07
CCDC124	1.350114	0.897982	-0.45213	5.44E-07
CCDC130	1.026553	0.646699	-0.37985	5.44E-07
CCDC142	1.302961	0.932529	-0.37043	5.44E-07
CCDC88B	0.962783	0.608432	-0.35435	5.44E-07
CCS	1.11463	0.775709	-0.33892	5.44E-07
CD151	1.803979	1.429331	-0.37465	5.44E-07
CD37	1.466708	1.1307	-0.33601	5.44E-07
CD52	2.244208	1.682509	-0.5617	5.44E-07
CD63	2.202573	1.825939	-0.37663	5.44E-07

CD74	2.669854	2.130565	-0.53929	5.44E-07
CD81	2.015438	1.624413	-0.39102	5.44E-07
CDC34	1.008697	0.648084	-0.36061	5.44E-07
CDC37	1.46011	1.089602	-0.37051	5.44E-07
CDK10	1.177747	0.804771	-0.37298	5.44E-07
CDK5RAP	1.354116	0.993743	-0.36037	5.44E-07
CDKL5	0.133117	0.610964	0.477847	1.21E-05
CEMP1	0.883828	0.521671	-0.36216	5.44E-07
CFD	1.735945	1.330732	-0.40521	5.44E-07
CFL1	2.002381	1.616841	-0.38554	5.44E-07
CHCHD1	1.122699	0.792612	-0.33009	5.44E-07
CHCHD2	1.309773	0.969946	-0.33983	5.44E-07
CHMP1A	1.242052	0.920097	-0.32196	5.44E-07
CHMP2A	1.608953	1.225687	-0.38327	5.44E-07
CHPF	1.142287	0.808331	-0.33396	5.44E-07
CIB1	1.61415	1.122227	-0.49192	5.44E-07
CIRBP	2.11962	1.768512	-0.35111	5.44E-07
CKB	1.370608	0.900407	-0.4702	5.44E-07
CKS2	1.221292	0.873673	-0.34762	5.44E-07
CLASRP	1.122079	0.785477	-0.3366	5.44E-07
CLC	0.260444	0.697204	0.43676	1.21E-05
CLDN3	0.918627	0.583136	-0.33549	5.44E-07
CLDN5	1.457222	1.092379	-0.36484	5.44E-07
CLEC3B	1.73829	1.313361	-0.42493	5.44E-07
CLIC3	1.539609	1.021516	-0.51809	5.44E-07
CLPP	1.170163	0.810059	-0.3601	5.44E-07
CLTA	1.569514	1.18795	-0.38156	5.44E-07
CLTB	1.246654	0.895003	-0.35165	5.44E-07
CNKSR1	0.883202	0.519797	-0.36341	5.44E-07
CNTN1	0.379525	0.701529	0.322004	1.21E-05
COA3	1.366982	0.864137	-0.50284	5.44E-07
COL6A2	1.6367	1.250477	-0.38622	5.44E-07
COMMD4	0.924913	0.588624	-0.33629	5.44E-07
COMTD1	0.789518	0.379702	-0.40982	5.44E-07
COPE	1.320467	0.890523	-0.42994	5.44E-07
CORO7	1.227767	0.842688	-0.38508	5.44E-07
COX14	1.434696	1.091908	-0.34279	5.44E-07
COX17	1.459812	0.929752	-0.53006	5.44E-07
COX4I1	1.909887	1.400971	-0.50892	5.44E-07
COX4I2	1.10175	0.648584	-0.45317	5.44E-07
COX5B	1.850159	1.465088	-0.38507	5.44E-07
COX6A1	1.30126	0.681573	-0.61969	5.44E-07
COX6B1	1.811232	1.379593	-0.43164	5.44E-07
COX7C	1.958279	1.61111	-0.34717	5.44E-07
COX8A	2.01169	1.432812	-0.57888	5.44E-07
CPSF3L	1.162198	0.7858	-0.3764	5.44E-07
CPT1B	1.277218	0.920474	-0.35674	5.44E-07
CRACR2B	0.956461	0.542242	-0.41422	5.44E-07
CRIP1	1.771916	1.140417	-0.6315	5.44E-07
CRIP2	1.707718	1.163664	-0.54405	5.44E-07
CROCC	0.786144	0.462794	-0.32335	5.44E-07
CSF3	0.675195	1.031423	0.356228	4.27E-05
CST3	2.109319	1.757023	-0.3523	5.44E-07
CST6	1.057598	0.676581	-0.38102	5.44E-07
CTBP1	1.415148	1.061736	-0.35341	5.44E-07
CTDSP1	1.423402	1.095342	-0.32806	5.44E-07

CTSD	2.363393	1.839887	-0.52351	5.44E-07
CTSH	2.067909	1.703289	-0.36462	5.44E-07
CTSZ	1.948819	1.566721	-0.3821	5.44E-07
CUTA	1.541681	1.16091	-0.38077	5.44E-07
CXCL8	1.12055	1.466515	0.345965	1.21E-05
CXCR1	0.290708	0.628362	0.337654	1.21E-05
CXCR2	0.318045	0.712489	0.394443	1.21E-05
CYBA	1.958196	1.54425	-0.41395	5.44E-07
CYHR1	1.00683	0.644545	-0.36229	5.44E-07
CYP1B1	0.77636	1.174608	0.398248	1.21E-05
CYP27A1	1.509124	1.170996	-0.33813	5.44E-07
CYP7B1	0.28837	0.636186	0.347816	1.21E-05
CYSTM1	1.590156	1.162713	-0.42744	5.44E-07
D2HGDH	1.002802	0.601542	-0.40126	5.44E-07
DAD1	1.852877	1.503156	-0.34972	5.44E-07
DAPK3	1.15639	0.831628	-0.32476	5.44E-07
DBI	1.899025	1.493809	-0.40522	5.44E-07
DCTPP1	1.006774	0.676341	-0.33043	5.44E-07
DCUN1D3	0.603973	0.942263	0.33829	1.21E-05
DCXR	1.325208	0.921303	-0.4039	5.44E-07
DDAH2	1.41655	1.074538	-0.34201	5.44E-07
DDR2	0.446229	0.881113	0.434884	1.21E-05
DDRGK1	1.31147	0.978108	-0.33336	5.44E-07
DEFB1	0.516358	0.981855	0.465497	1.21E-05
DES	1.214838	0.832243	-0.38259	5.44E-07
DGCR6	0.883927	0.549105	-0.33482	5.44E-07
DGCR6L	1.039664	0.683707	-0.35596	5.44E-07
DGUOK	1.194714	0.866017	-0.3287	5.44E-07
DMPK	1.182677	0.798299	-0.38438	5.44E-07
DNAJC4	1.045504	0.695828	-0.34968	5.44E-07
DNPH1	1.157555	0.785314	-0.37224	5.44E-07
DOCK5	0.505418	0.837302	0.331884	1.21E-05
DOHH	0.758982	0.428616	-0.33037	5.44E-07
DOK2	1.26563	0.878962	-0.38667	5.44E-07
DPM2	0.996713	0.664645	-0.33207	5.44E-07
DPM3	1.444828	0.956847	-0.48798	5.44E-07
DPP7	1.429937	1.032561	-0.39738	5.44E-07
DPP8	0.596943	0.96085	0.363907	1.21E-05
DRAP1	1.405672	0.997929	-0.40774	5.44E-07
DUS1L	1.122876	0.788978	-0.3339	5.44E-07
DUSP23	1.200799	0.720798	-0.48	5.44E-07
DVL1	1.108556	0.722594	-0.38596	5.44E-07
DYNLL1	1.670494	1.180664	-0.48983	5.44E-07
E4F1	0.826298	0.498572	-0.32773	5.44E-07
ECH1	1.610394	1.230668	-0.37973	5.44E-07
ECHDC2	1.170409	0.758858	-0.41155	5.44E-07
ECI1	1.049146	0.683702	-0.36544	5.44E-07
EDARADD	1.210408	0.823621	-0.38679	5.44E-07
EDF1	1.885263	1.407287	-0.47798	5.44E-07
EEF1D	1.489866	0.922045	-0.56782	5.44E-07
EEF1G	1.285723	0.925015	-0.36071	5.44E-07
EEF2	2.110056	1.763606	-0.34645	5.44E-07
EFNA1	1.430242	1.071522	-0.35872	5.44E-07
EGFL7	1.407348	0.922964	-0.48438	5.44E-07
EGFR	0.724307	1.059416	0.335108	1.21E-05
EIF2AK2	0.542013	0.981881	0.439867	1.21E-05

https://doi.org/10.5808/GI.2019.17.1.e2

EIF3G	1.635581	1.26988	-0.3657	5.44E-07
EIF3K	1.524073	1.113882	-0.41019	5.44E-07
EIF4EBP1	1.053806	0.678746	-0.37506	5.44E-07
EIF6	1.41156	1.081393	-0.33017	5.44E-07
ELMO3	0.758329	0.435817	-0.32251	5.44E-07
EMP3	1.415424	0.989631	-0.42579	5.44E-07
ENKD1	0.860601	0.489634	-0.37097	5.44E-07
ENTHD2	0.879072	0.495284	-0.38379	5.44E-07
ENTPD5	0.448965	0.779293	0.330328	1.21E-05
EPN1	1.315634	0.95035	-0.36528	5.44E-07
EPOR	0.913672	0.571311	-0.34236	5.44E-07
EPS8L1	0.883301	0.546072	-0.33723	5.44E-07
EPS8L2	1.399905	0.965752	-0.43415	5.44E-07
EREG	0.352129	0.727597	0.375468	1.21E-05
ERH	1.586884	1.255503	-0.33138	5.44E-07
ERN1	0.569674	0.925425	0.35575	1.21E-05
ERP29	1.504699	1.16143	-0.34327	5.44E-07
ETFB	1.392047	0.889283	-0.50276	5.44E-07
EVA1B	1.026237	0.62954	-0.3967	5.44E-07
EVPL	0.996909	0.627353	-0.36956	5.44E-07
EXD3	0.856378	0.533093	-0.32328	5.44E-07
EXOC6B	0.467427	0.855358	0.387931	1.21E-05
EXOSC4	0.939749	0.591199	-0.34855	5.44E-07
FABP4	1.834941	1.486143	-0.3488	5.44E-07
FADS3	1.137198	0.763246	-0.37395	5.44E-07
FAM110A	0.951864	0.609323	-0.34254	5.44E-07
FAM127A	1.519663	1.124773	-0.39489	5.44E-07
FAM127B	1.259186	0.779961	-0.47922	5.44E-07
FAM129B	1.56234	1.205918	-0.35642	5.44E-07
FAM193B	1.139827	0.742719	-0.39711	5.44E-07
FAM195B	1.26956	0.880914	-0.38865	5.44E-07
FAM3A	1.054149	0.661658	-0.39249	5.44E-07
FAM50A	1.292895	0.928139	-0.36476	5.44E-07
FAM63B	0.308993	0.667436	0.358444	1.21E-05
FAM96B	1.375665	0.949526	-0.42614	5.44E-07
FASTK	1.124789	0.684716	-0.44007	5.44E-07
FAU	1.808328	1.276718	-0.53161	5.44E-07
FBL	1.438991	1.025079	-0.41391	5.44E-07
FBP1	1.711391	1.321593	-0.3898	5.44E-07
FBXL15	0.737084	0.408002	-0.32908	5.44E-07
FBXW5	1.318725	0.932035	-0.38669	5.44E-07
FCGRT	1.646791	1.249006	-0.39778	5.44E-07
FDX1L	0.863813	0.475754	-0.38806	5.44E-07
FGA	0.177779	0.555975	0.378196	1.21E-05
FGF7	0.56864	0.951539	0.382899	1.21E-05
FGG	0.674339	1.265561	0.591222	1.21E-05
FHOD1	1.224942	0.884632	-0.34031	5.44E-07
FIS1	1.578546	1.172072	-0.40647	5.44E-07
FKBP2	1.39361	0.901359	-0.49225	5.44E-07
FKBP8	1.49508	1.014338	-0.48074	5.44E-07
FLYWCH2	0.972942	0.634712	-0.33823	5.44E-07
FOLR1	1.622377	1.289375	-0.333	5.44E-07
FTL	2.840933	2.185637	-0.6553	5.44E-07
FUOM	0.992421	0.548741	-0.44368	5.44E-07
FXYD5	1.558157	1.21424	-0.34392	5.44E-07
GABARAP	1.879582	1.459558	-0.42002	5.44E-07

https://doi.org/10.5808/GI.2019.17.1.e2

GADD45G	0.950128	0.600417	-0.34971	5.44E-07
GADD45G	1.156777	0.762025	-0.39475	5.44E-07
GALNT5	0.602513	0.929738	0.327225	1.21E-05
GAPDH	1.713805	1.365893	-0.34791	5.44E-07
GAS6	1.63103	1.203945	-0.42709	5.44E-07
GCHFR	1.296759	0.96258	-0.33418	5.44E-07
GDPD3	0.710183	0.379455	-0.33073	5.44E-07
GIPC1	1.325278	0.958119	-0.36716	5.44E-07
GMFG	1.679327	1.33702	-0.34231	5.44E-07
GNB2	1.544026	1.116575	-0.42745	5.44E-07
GNB2L1	2.183623	1.722484	-0.46114	5.44E-07
GNG11	1.729968	1.317407	-0.41256	5.44E-07
GNG5	1.501163	1.055483	-0.44568	5.44E-07
GNLY	1.097536	0.751381	-0.34615	5.44E-07
GPR15	0.196837	0.609904	0.413067	1.21E-05
GPRIN3	0.369489	0.695414	0.325925	1.21E-05
GPS1	1.17997	0.828543	-0.35143	5.44E-07
GPSM1	0.975066	0.59226	-0.38281	5.44E-07
GPX1	1.698507	1.149835	-0.54867	5.44E-07
GPX4	1.766243	1.326319	-0.43992	5.44E-07
GRN	2.005509	1.575456	-0.43005	5.44E-07
GSDMD	1.292635	0.879506	-0.41313	5.44E-07
GSN	1.882653	1.544254	-0.3384	5.44E-07
GSTP1	1.994923	1.496165	-0.49876	5.44E-07
GTF2I	1.01526	1.394101	0.378842	1.21E-05
GTF3C3	0.73395	1.059447	0.325497	1.21E-05
GUK1	1.647264	1.307903	-0.33936	5.44E-07
GYPC	1.396259	1.051567	-0.34469	5.44E-07
HACD2	0.821565	1.270309	0.448744	1.21E-05
HAS2	0.601761	1.004055	0.402295	1.21E-05
HAUS7	0.865877	0.522462	-0.34341	5.44E-07
HBA2	0.935842	0.521387	-0.41445	5.44E-07
HCFC1R1	1.280726	0.896577	-0.38415	5.44E-07
HCST	1.416683	0.984255	-0.43243	5.44E-07
HDAC10	0.78697	0.432312	-0.35466	5.44E-07
HEXDC	0.947426	0.569987	-0.37744	5.44E-07
HIGD1B	1.220643	0.732466	-0.48818	5.44E-07
HIGD2A	1.474239	1.009984	-0.46425	5.44E-07
HINT1	1.807688	1.467504	-0.34018	5.44E-07
HINT2	1.147381	0.76598	-0.3814	5.44E-07
HLA-A	2.051321	1.581603	-0.46972	5.44E-07
HLA-B	2.37656	1.891062	-0.4855	5.44E-07
HLA-C	2.234197	1.868582	-0.36561	5.44E-07
HLA-DMA	1.845496	1.518876	-0.32662	5.44E-07
HLA-DRA	2.603524	2.278139	-0.32538	5.44E-07
HLA-DRB1	2.177798	1.686846	-0.49095	5.44E-07
HLA-DRB5	1.643108	1.232698	-0.41041	5.44E-07
HLA-F	1.612025	1.260023	-0.352	5.44E-07
HMBOX1	0.323247	0.864832	0.541585	1.21E-05
HMG20B	1.276802	0.94175	-0.33505	5.44E-07
HMHA1	1.165191	0.814918	-0.35027	5.44E-07
HOOK2	1.024218	0.687664	-0.33655	5.44E-07
HPN	0.872859	0.526787	-0.34607	5.44E-07
HRAS	1.035316	0.544789	-0.49053	5.44E-07
HSPB1	1.91551	1.35801	-0.5575	5.44E-07
HSPBP1	0.907899	0.564126	-0.34377	5.44E-07

ICAM5	0.943668	0.60589	-0.33778	5.44E-07
ID1	1.76399	1.37051	-0.39348	5.44E-07
IDH3G	1.156673	0.78608	-0.37059	5.44E-07
IFI27	2.105347	1.613378	-0.49197	5.44E-07
IFI27L2	1.573999	1.032188	-0.54181	5.44E-07
IFI30	2.205636	1.773781	-0.43185	5.44E-07
IFI6	1.836293	1.34956	-0.48673	5.44E-07
IFITM2	1.740385	1.347212	-0.39317	5.44E-07
IFITM3	2.113201	1.627516	-0.48569	5.44E-07
IFT43	1.260805	0.8208	-0.44001	5.44E-07
IGSF8	1.037954	0.71472	-0.32323	5.44E-07
IL11RA	0.932921	0.600789	-0.33213	5.44E-07
IL6	1.225023	1.606523	0.3815	1.21E-05
IMP3	1.298431	0.957815	-0.34062	5.44E-07
INF2	1.265967	0.938298	-0.32767	5.44E-07
INHBA	0.735511	1.107369	0.371859	1.21E-05
IRF3	1.204728	0.798289	-0.40644	5.44E-07
IRF7	0.937445	0.586125	-0.35132	5.44E-07
ISG15	1.385659	0.929047	-0.45661	5.44E-07
ISOC2	1.005876	0.661152	-0.34472	5.44E-07
ITPA	1.179242	0.827027	-0.35221	5.44E-07
JMJD8	1.183016	0.827957	-0.35506	5.44E-07
JOSD2	0.989326	0.565567	-0.42376	5.44E-07
JTB	1.414666	1.059867	-0.3548	5.44E-07
KANK3	1.034726	0.700705	-0.33402	5.44E-07
KAT2A	1.116524	0.791271	-0.32525	5.44E-07
KIFC2	0.957982	0.540853	-0.41713	5.44E-07
KIFC3	1.172277	0.793599	-0.37868	5.44E-07
KPNA5	0.392058	0.715928	0.32387	1.21E-05
KRT18	1.606263	1.278817	-0.32745	5.44E-07
KRT19	1.689622	1.262068	-0.42755	5.44E-07
KRTCAP2	1.553693	1.031447	-0.52225	5.44E-07
KRTCAP3	0.944302	0.609145	-0.33516	5.44E-07
LAGE3	0.889276	0.433092	-0.45618	5.44E-07
LAMTOR4	1.615459	1.191888	-0.42357	5.44E-07
LAMTOR5	1.514443	1.081666	-0.43278	5.44E-07
LAT	0.919798	0.548181	-0.37162	5.44E-07
LGALS1	2.257071	1.677852	-0.57922	5.44E-07
LGALS3B	1.868657	1.464529	-0.40413	5.44E-07
LIMS2	1.353931	0.999266	-0.35466	5.44E-07
LLGL2	1.217162	0.855306	-0.36186	5.44E-07
LMBRD2	0.283195	0.64065	0.357454	1.21E-05
LMF2	1.224592	0.860218	-0.36437	5.44E-07
LNPEP	0.286208	0.694722	0.408514	1.21E-05
LRFN4	0.697171	0.370298	-0.32687	5.44E-07
LRRC75B	0.617565	0.26543	-0.35213	5.44E-07
LSM3	1.265558	0.926484	-0.33907	5.44E-07
LSM7	1.41217	0.877969	-0.5342	5.44E-07
LSR	1.243652	0.907155	-0.3365	5.44E-07
LTBP3	1.421185	1.061153	-0.36003	5.44E-07
LTBP4	1.67257	1.334006	-0.33856	5.44E-07
LUC7L	1.234278	0.840639	-0.39364	5.44E-07
LY6E	1.835047	1.429611	-0.40544	5.44E-07
LYPLA2	0.916554	0.577065	-0.33949	5.44E-07
LZTS2	1.089139	0.703057	-0.38608	5.44E-07
MACROD1	0.901737	0.565189	-0.33655	5.44E-07

https://doi.org/10.5808/GI.2019.17.1.e2

MALSU1	1.10393	0.769152	-0.33478	5.44E-07
MAN1A2	0.53857	0.892406	0.353836	1.21E-05
MAP1LC3	1.14014	0.816465	-0.32367	5.44E-07
MAP7D1	1.305064	0.980922	-0.32414	5.44E-07
MAPK15	0.785509	0.432915	-0.35259	5.44E-07
MAPK3	1.234314	0.8652	-0.36911	5.44E-07
MAPK8IP3	1.032144	0.700691	-0.33145	5.44E-07
MARCO	1.713341	1.373037	-0.3403	5.44E-07
MBD3	1.194604	0.773594	-0.42101	5.44E-07
MDK	1.179405	0.806018	-0.37339	5.44E-07
MDP1	1.545144	1.126777	-0.41837	5.44E-07
MEA1	1.190133	0.841434	-0.3487	5.44E-07
MFSD10	1.33757	0.92153	-0.41604	5.44E-07
MGAT4B	1.261464	0.937537	-0.32393	5.44E-07
MIA	1.562409	1.144894	-0.41752	5.44E-07
MIB2	0.8755	0.48901	-0.38649	5.44E-07
MIEN1	1.226994	0.855432	-0.37156	5.44E-07
MIF	1.881353	1.291278	-0.59008	5.44E-07
MIIP	0.952312	0.584555	-0.36776	5.44E-07
MINOS1	1.696823	1.356134	-0.34069	5.44E-07
MLST8	0.919843	0.591524	-0.32832	5.44E-07
MMRN1	0.664502	1.083491	0.418988	1.21E-05
MOB2	1.052041	0.690468	-0.36157	5.44E-07
MOSPD3	0.928508	0.575707	-0.3528	5.44E-07
MPG	1.197619	0.788835	-0.40878	5.44E-07
MPLKIP	1.108525	0.768997	-0.33953	5.44E-07
MPST	1.243915	0.893907	-0.35001	5.44E-07
MRPL12	1.060821	0.698817	-0.362	5.44E-07
MRPL14	1.484898	1.00298	-0.48192	5.44E-07
MRPL20	1.583778	1.208799	-0.37498	5.44E-07
MRPL21	1.176832	0.803673	-0.37316	5.44E-07
MRPL23	1.322675	0.870862	-0.45181	5.44E-07
MRPL27	1.298199	0.910811	-0.38739	5.44E-07
MRPL28	1.149865	0.803124	-0.34674	5.44E-07
MRPL38	1.024559	0.663344	-0.36122	5.44E-07
MRPL4	1.031044	0.616407	-0.41464	5.44E-07
MRPL41	1.406619	0.810615	-0.596	5.44E-07
MRPL51	1.599581	1.252784	-0.3468	5.44E-07
MRPL54	1.288356	0.940592	-0.34776	5.44E-07
MRPL55	1.024464	0.575148	-0.44932	5.44E-07
MRPS12	0.888739	0.548708	-0.34003	5.44E-07
MRPS2	0.998368	0.664041	-0.33433	5.44E-07
MRPS26	1.004416	0.633524	-0.37089	5.44E-07
MRPS34	1.20684	0.766727	-0.44011	5.44E-07
MS4A15	1.152305	0.809028	-0.34328	5.44E-07
MS4A6E	0.954689	0.540574	-0.41412	5.44E-07
MSLN	1.387114	0.988087	-0.39903	5.44E-07
MUC1	1.898011	1.542695	-0.35532	5.44E-07
MVD	1.088012	0.710657	-0.37736	5.44E-07
MVP	1.512945	1.184766	-0.32818	5.44E-07
MYDGF	1.535285	1.213075	-0.32221	5.44E-07
MYEOV2	1.610845	1.202209	-0.40864	5.44E-07
MYL6	2.267636	1.730656	-0.53698	5.44E-07
MYL9	1.821528	1.451419	-0.37011	5.44E-07
MYO9A	0.318143	0.690448	0.372306	1.21E-05
MZT2A	1.170915	0.761418	-0.4095	5.44E-07

MZT2B	1.058062	0.67633	-0.38173	5.44E-07
NAA10	1.113455	0.731766	-0.38169	5.44E-07
NACA	1.713573	1.30992	-0.40365	5.44E-07
NAT14	0.843866	0.480436	-0.36343	5.44E-07
NAT9	1.006482	0.663643	-0.34284	5.44E-07
NCLN	1.041417	0.707775	-0.33364	5.44E-07
NCOA2	0.478903	0.825648	0.346745	1.21E-05
NCOA7	1.229627	1.556277	0.32665	1.21E-05
NDUFA11	1.489914	1.091278	-0.39864	5.44E-07
NDUFA13	1.786166	1.287294	-0.49887	5.44E-07
NDUFA2	1.509755	1.161306	-0.34845	5.44E-07
NDUFA4	1.005728	0.63135	-0.37438	5.44E-07
NDUFA7	1.266945	0.793617	-0.47333	5.44E-07
NDUFAB1	1.514604	1.094647	-0.41996	5.44E-07
NDUFAF3	1.260826	0.909112	-0.35171	5.44E-07
NDUFB10	1.379099	0.973237	-0.40586	5.44E-07
NDUFB11	1.274036	0.930028	-0.34401	5.44E-07
NDUFB2	1.797189	1.327681	-0.46951	5.44E-07
NDUFB7	1.612452	1.085363	-0.52709	5.44E-07
NDUFS3	1.297321	0.917128	-0.38019	5.44E-07
NDUFS5	1.876765	1.434934	-0.44183	5.44E-07
NDUFS6	1.515156	1.12269	-0.39247	5.44E-07
NDUFS7	1.391531	0.953741	-0.43779	5.44E-07
NDUFS8	1.229227	0.838702	-0.39053	5.44E-07
NDUFV1	1.367274	0.980804	-0.38647	5.44E-07
NDUFV2	0.925139	0.593036	-0.3321	5.44E-07
NELFA	1.081879	0.680343	-0.40154	5.44E-07
NELFB	1.053381	0.727627	-0.32575	5.44E-07
NFKBIL1	0.840247	0.516677	-0.32357	5.44E-07
NHLRC2	0.35632	0.69199	0.33567	1.21E-05
NHP2	1.034384	0.653681	-0.3807	5.44E-07
NHSL2	0.201664	0.570745	0.36908	1.21E-05
NME1	1.690121	1.247682	-0.44244	5.44E-07
NME3	1.206693	0.65188	-0.55481	5.44E-07
NMRAL1	0.998077	0.65327	-0.34481	5.44E-07
NOL3	0.965358	0.5957	-0.36966	5.44E-07
NOP10	1.719264	1.339664	-0.3796	5.44E-07
NOSIP	1.108918	0.768295	-0.34062	5.44E-07
NPC2	2.247446	1.899708	-0.34774	5.44E-07
NPDC1	1.157757	0.799359	-0.3584	5.44E-07
NR1H2	1.326644	1.003612	-0.32303	5.44E-07
NR4A3	0.916292	1.354153	0.437861	1.21E-05
NT5C	1.018108	0.639967	-0.37814	5.44E-07
NTHL1	0.812691	0.453888	-0.3588	5.44E-07
NTRK2	0.27481	0.5997	0.32489	1.21E-05
NUBP2	1.092488	0.667013	-0.42547	5.44E-07
NUCB1	1.660437	1.331339	-0.3291	5.44E-07
NUDT22	0.969208	0.625209	-0.344	5.44E-07
NUPR1	1.717411	1.283072	-0.43434	5.44E-07
OAZ1	2.105673	1.626196	-0.47948	5.44E-07
OBSL1	1.124886	0.781497	-0.34339	5.44E-07
OCLM	0.144163	0.476549	0.332385	1.21E-05
ODF3B	1.207893	0.766647	-0.44125	5.44E-07
OGFR	1.090092	0.682302	-0.40779	5.44E-07
OST4	1.890628	1.483645	-0.40698	5.44E-07
OXLD1	0.9185	0.527721	-0.39078	5.44E-07

PALM3	0.635599	0.298539	-0.33706	5.44E-07
PANK3	0.395355	0.760449	0.365094	1.21E-05
PARP10	1.216959	0.843259	-0.3737	5.44E-07
PCBP4	0.948983	0.617263	-0.33172	5.44E-07
PCOLCE	1.265347	0.901918	-0.36343	5.44E-07
PDLIM2	1.386712	1.005715	-0.381	5.44E-07
PDLIM7	1.273189	0.905423	-0.36777	5.44E-07
PDZK1IP1	1.205442	0.862615	-0.34283	5.44E-07
PEBP4	1.676196	1.322163	-0.35403	5.44E-07
PEF1	1.219722	0.865852	-0.35387	5.44E-07
PEX16	0.853533	0.489878	-0.36366	5.44E-07
PFDN2	1.489859	1.099023	-0.39084	5.44E-07
PFDN5	1.993899	1.62148	-0.37242	5.44E-07
PFN1	1.862549	1.402837	-0.45971	5.44E-07
PGAP3	1.049164	0.724478	-0.32469	5.44E-07
PGLS	1.221072	0.891801	-0.32927	5.44E-07
PHPT1	1.25032	0.807589	-0.44273	5.44E-07
PIDD1	0.705814	0.344688	-0.36113	5.44E-07
PIGQ	1.081819	0.735291	-0.34653	5.44E-07
PILRB	1.225548	0.897281	-0.32827	5.44E-07
PKN1	1.392822	1.066056	-0.32677	5.44E-07
PLA2G1B	1.210309	0.828271	-0.38204	5.44E-07
PLAC9	1.325958	0.867769	-0.45819	5.44E-07
PLD3	1.776409	1.431051	-0.34536	5.44E-07
PLEKHF1	0.771325	0.431081	-0.34024	5.44E-07
PLEKHH3	0.978862	0.590425	-0.38844	5.44E-07
PMF1	1.285939	0.942391	-0.34355	5.44E-07
PMM1	1.16666	0.784903	-0.38176	5.44E-07
PNKD	1.260651	0.882777	-0.37787	5.44E-07
PNKP	1.07564	0.749336	-0.3263	5.44E-07
PNPLA2	1.432698	1.071497	-0.3612	5.44E-07
PNPLA6	1.446655	1.093913	-0.35274	5.44E-07
POLD4	1.191645	0.747487	-0.44416	5.44E-07
POLE4	1.268972	0.940965	-0.32801	5.44E-07
POLR2H	1.173702	0.831405	-0.3423	5.44E-07
POLR2I	0.916043	0.584045	-0.332	5.44E-07
POP5	1.076942	0.753096	-0.32385	5.44E-07
PPAN-P2R	0.987187	0.657086	-0.3301	5.44E-07
PPDPF	1.603121	1.09675	-0.50637	5.44E-07
PPIB	1.881295	1.491792	-0.3895	5.44E-07
PPM1L	0.222395	0.59862	0.376225	1.21E-05
PPP1R12C	1.22288	0.795647	-0.42723	5.44E-07
PPP1R13L	1.039189	0.682124	-0.35707	5.44E-07
PPP1R16A	0.913637	0.501186	-0.41245	5.44E-07
PPP4C	1.392089	1.029965	-0.36212	5.44E-07
PQBP1	1.410297	1.042905	-0.36739	5.44E-07
PQLC1	1.148948	0.822476	-0.32647	5.44E-07
PRDX1	2.019856	1.67594	-0.34392	5.44E-07
PRDX5	1.776107	1.314663	-0.46144	5.44E-07
PRELID1	1.000542	0.674575	-0.32597	5.44E-07
PRKAR2A	0.653308	1.023854	0.370546	1.21E-05
PRKCDBP	1.278062	0.844354	-0.43371	5.44E-07
PRKCSH	1.579099	1.233842	-0.34526	5.44E-07
PRMT1	1.352351	1.00825	-0.3441	5.44E-07
PRODH	1.185406	0.827075	-0.35833	5.44E-07
PROK2	0.194195	0.546777	0.352581	1.21E-05

PRR14	1.052582	0.71821	-0.33437	5.44E-07
PRSS8	1.385417	0.979392	-0.40602	5.44E-07
PSENEN	1.293034	0.924692	-0.36834	5.44E-07
PSMA7	1.659181	1.279031	-0.38015	5.44E-07
PSMB10	1.392803	0.967323	-0.42548	5.44E-07
PSMB3	1.48776	1.098418	-0.38934	5.44E-07
PSMB4	1.73487	1.370649	-0.36422	5.44E-07
PSMB6	1.468092	1.089637	-0.37845	5.44E-07
PSMB9	1.46611	1.12331	-0.3428	5.44E-07
PSMD4	1.211956	0.888689	-0.32327	5.44E-07
PSME2	1.141943	0.752988	-0.38895	5.44E-07
PTGDS	1.99374	1.628232	-0.36551	5.44E-07
PTGS2	1.082073	1.48007	0.397997	1.21E-05
PTMS	1.446949	1.08894	-0.35801	5.44E-07
PTOV1	1.085107	0.719801	-0.36531	5.44E-07
PTPRCAP	1.170076	0.621623	-0.54845	5.44E-07
PTRHD1	1.323298	0.941313	-0.38198	5.44E-07
PUF60	1.243696	0.911378	-0.33232	5.44E-07
PVRIG	0.853604	0.50323	-0.35037	5.44E-07
PWWP2B	0.95532	0.619176	-0.33614	5.44E-07
PYCARD	1.35713	0.938601	-0.41853	5.44E-07
QTRT1	1.075263	0.703605	-0.37166	5.44E-07
RAB11B	1.455708	1.04828	-0.40743	5.44E-07
RAB17	0.981749	0.653123	-0.32863	5.44E-07
RAB24	1.106892	0.691867	-0.41502	5.44E-07
RAB40C	0.935425	0.60564	-0.32979	5.44E-07
RABAC1	1.579014	1.159702	-0.41931	5.44E-07
RABEP2	0.800994	0.456423	-0.34457	5.44E-07
RAD54L2	0.528163	0.869522	0.341359	1.21E-05
RAMP2	1.600941	1.267813	-0.33313	5.44E-07
RAMP3	1.346952	1.012955	-0.334	5.44E-07
RANGRF	1.064121	0.739303	-0.32482	5.44E-07
RARRES2	1.802842	1.385513	-0.41733	5.44E-07
RARRES3	1.556317	1.196987	-0.35933	5.44E-07
RASSF7	1.332149	0.719613	-0.61254	5.44E-07
RBCK1	1.23817	0.877162	-0.36101	5.44E-07
RBM42	1.314713	0.926531	-0.38818	5.44E-07
RBP1	1.054222	0.722427	-0.33179	5.44E-07
RC3H2	0.436342	0.761522	0.325179	1.21E-05
RCN3	0.966162	0.616169	-0.34999	5.44E-07
RDH5	1.613051	1.21156	-0.40149	5.44E-07
REL	0.449746	0.899385	0.449639	1.21E-05
RETN	1.327204	0.920233	-0.40697	5.44E-07
RFXANK	1.144581	0.805284	-0.3393	5.44E-07
RGL3	1.089554	0.745967	-0.34359	5.44E-07
RHBDD2	1.495159	1.163989	-0.33117	5.44E-07
RHOC	1.504645	1.138503	-0.36614	5.44E-07
RHOT2	1.293176	0.900791	-0.39239	5.44E-07
RNASE1	2.240185	1.819661	-0.42052	5.44E-07
RNASEK	1.828564	1.475174	-0.35339	5.44E-07
RNF181	1.307348	0.916231	-0.39112	5.44E-07
RNF24	0.49938	0.848426	0.349046	1.21E-05
RNH1	1.548152	1.150045	-0.39811	5.44E-07
RNPEPL1	1.196652	0.845213	-0.35144	5.44E-07
ROGDI	1.084049	0.697126	-0.38692	5.44E-07
RPL11	2.264101	1.878604	-0.3855	5.44E-07

RPL13A	1.596155	1.230509	-0.36565	5.44E-07
RPL18	1.69945	1.268276	-0.43117	5.44E-07
RPL19	1.73124	1.287429	-0.44381	5.44E-07
RPL23A	1.067115	0.620664	-0.44645	5.44E-07
RPL26	1.596297	1.041442	-0.55485	5.44E-07
RPL27	1.948079	1.440542	-0.50754	5.44E-07
RPL28	1.956459	1.224502	-0.73196	5.44E-07
RPL29	1.635724	1.179812	-0.45591	5.44E-07
RPL31	2.203544	1.881576	-0.32197	5.44E-07
RPL34	1.925526	1.544261	-0.38126	5.44E-07
RPL35	2.091507	0.991533	-1.09997	5.44E-07
RPL35A	2.438379	1.932752	-0.50563	5.44E-07
RPL36	1.968693	1.48379	-0.4849	5.44E-07
RPL36AL	2.102832	1.655266	-0.44757	5.44E-07
RPL39	1.16817	0.80277	-0.3654	5.44E-07
RPL7A	1.416476	0.939805	-0.47667	5.44E-07
RPL8	2.274599	1.71829	-0.55631	5.44E-07
RPLP1	2.337686	1.539823	-0.79786	5.44E-07
RPLP2	2.623598	1.75181	-0.87179	5.44E-07
RPS11	1.994871	1.559298	-0.43557	5.44E-07
RPS12	1.75709	1.004126	-0.75296	5.44E-07
RPS15A	2.10636	1.758473	-0.34789	5.44E-07
RPS16	2.092233	1.518272	-0.57396	5.44E-07
RPS19	1.820536	1.430973	-0.38956	5.44E-07
RPS19BP1	1.231397	0.761117	-0.47028	5.44E-07
RPS21	2.1772	1.83987	-0.33733	5.44E-07
RPS24	1.977922	1.359934	-0.61799	5.44E-07
RPS28	0.985237	0.541668	-0.44357	5.44E-07
RPS4X	2.039251	1.648242	-0.39101	5.44E-07
RPS4Y1	1.682644	1.35054	-0.3321	5.44E-07
RPS6KB2	0.991136	0.650016	-0.34112	5.44E-07
RPS7	0.889088	0.511782	-0.37731	5.44E-07
RPS8	1.828352	1.39114	-0.43721	5.44E-07
RPUSD1	0.715226	0.368928	-0.3463	5.44E-07
RRAS	1.591227	1.128879	-0.46235	5.44E-07
RSRP1	1.442837	1.103413	-0.33942	5.44E-07
RUVBL2	1.133906	0.754201	-0.37971	5.44E-07
S100A10	2.163782	1.711128	-0.45265	5.44E-07
S100A11	2.082999	1.649117	-0.43388	5.44E-07
S100A12	0.574043	0.977039	0.402996	1.21E-05
S100A13	1.712501	1.302882	-0.40962	5.44E-07
S100A14	1.498542	1.106383	-0.39216	5.44E-07
S100A4	2.519145	1.929016	-0.59013	5.44E-07
S100A6	2.590091	1.853254	-0.73684	5.44E-07
S1PR4	0.852701	0.515738	-0.33696	5.44E-07
SAC3D1	0.616649	0.27361	-0.34304	5.44E-07
SAP25	0.671528	0.340722	-0.33081	5.44E-07
SAT2	1.255296	0.906165	-0.34913	5.44E-07
SCAND1	1.161179	0.632438	-0.52874	5.44E-07
SCGB1A1	2.223526	1.76441	-0.45912	0.000297
SCGB3A1	1.802801	1.227313	-0.57549	5.44E-07
SCGB3A2	2.333292	2.009274	-0.32402	1.04E-06
SCRN2	1.048839	0.692138	-0.3567	5.44E-07
SDE2	0.648183	1.013047	0.364864	1.21E-05
SDR39U1	1.155225	0.709736	-0.44549	5.44E-07
SEC61B	1.666212	0.985479	-0.68073	5.44E-07

SELE	0.564013	0.925975	0.361962	1.21E-05
SELENBP	1.799456	1.403056	-0.3964	5.44E-07
SEMA3B	1.422578	0.971014	-0.45156	5.44E-07
SEPW1	1.714329	1.243154	-0.47118	5.44E-07
SERPINE1	0.819436	1.199968	0.380532	1.21E-05
SF3A2	1.055574	0.707376	-0.3482	5.44E-07
SF3B5	1.522549	1.115805	-0.40674	5.44E-07
SFTA2	1.883168	1.434808	-0.44836	5.44E-07
SFTPA1	2.847935	2.490832	-0.3571	5.44E-07
SFTPA2	2.936417	2.573137	-0.36328	5.44E-07
SFTPB	2.553771	2.191749	-0.36202	5.44E-07
SFTPC	3.293278	2.40338	-0.8899	5.44E-07
SGMS2	0.97186	1.298313	0.326453	1.21E-05
SGPP2	0.701003	1.024413	0.323411	1.21E-05
SH2B1	1.090583	0.731222	-0.35936	5.44E-07
SH3BGRL	1.725603	1.242007	-0.4836	5.44E-07
SHARPIN	1.055858	0.647197	-0.40866	5.44E-07
SHC2	0.846654	0.469231	-0.37742	5.44E-07
SHFM1	1.856783	1.343742	-0.51304	5.44E-07
SIGIRR	1.105178	0.696652	-0.40853	5.44E-07
SIRT7	0.953107	0.612151	-0.34096	5.44E-07
SIVA1	1.354859	0.920861	-0.434	5.44E-07
SLC19A1	1.14772	0.791392	-0.35633	5.44E-07
SLC22A31	1.092283	0.718572	-0.37371	5.44E-07
SLC25A29	1.215101	0.806039	-0.40906	5.44E-07
SLC25A39	1.272584	0.878998	-0.39359	5.44E-07
SLC39A13	1.128696	0.794893	-0.3338	5.44E-07
SLC39A3	0.97246	0.633126	-0.33933	5.44E-07
SLC6A14	0.813572	1.195726	0.382154	1.21E-05
SLC7A2	0.636757	0.96597	0.329214	1.21E-05
SLC9A3R2	1.376223	1.029814	-0.34641	5.44E-07
SLFN5	0.760544	1.148388	0.387844	1.21E-05
SLPI	2.374571	2.043755	-0.33082	5.44E-07
SMUG1	0.941172	0.584686	-0.35649	5.44E-07
SNAPC2	0.889815	0.557188	-0.33263	5.44E-07
SNRNP35	1.16199	0.82738	-0.33461	5.44E-07
SNRNP70	1.687553	1.231333	-0.45622	5.44E-07
SNRPA	1.09529	0.758466	-0.33682	5.44E-07
SNRPD2	1.508078	1.110969	-0.39711	5.44E-07
SORBS3	1.4264	1.081054	-0.34535	5.44E-07
SPAG7	1.319034	0.948447	-0.37059	5.44E-07
SPG7	1.221626	0.898841	-0.32278	5.44E-07
SPINT2	1.722466	1.322147	-0.40032	5.44E-07
SPNS1	1.229679	0.804567	-0.42511	5.44E-07
SPON2	1.231456	0.840242	-0.39121	5.44E-07
SPSB2	0.865379	0.496481	-0.3689	5.44E-07
SPSB3	1.261106	0.699447	-0.56166	5.44E-07
SSNA1	1.167017	0.797563	-0.36945	5.44E-07
SSR4	1.735234	1.318874	-0.41636	5.44E-07
ST8SIA6	0.236291	0.675047	0.438756	1.21E-05
STRA13	1.174114	0.680341	-0.49377	5.44E-07
STUB1	1.28965	0.850124	-0.43953	5.44E-07
STX10	1.193514	0.829027	-0.36449	5.44E-07
STX8	1.311998	0.849108	-0.46289	5.44E-07
STXBP2	1.348864	0.992101	-0.35676	5.44E-07
SURF1	1.183245	0.840201	-0.34304	5.44E-07

SURF2	0.838679	0.494913	-0.34377	5.44E-07
SUSD2	1.67622	1.288537	-0.38768	5.44E-07
SYNGR2	1.547356	1.19148	-0.35588	5.44E-07
SYTL1	1.003935	0.663956	-0.33998	5.44E-07
TACSTD2	1.671438	1.337406	-0.33403	5.44E-07
TAF1C	1.206769	0.854716	-0.35205	5.44E-07
TAGLN	1.84421	1.459676	-0.38453	5.44E-07
TAZ	1.023345	0.680057	-0.34329	5.44E-07
TBC1D10C	1.035253	0.628418	-0.40683	5.44E-07
TBCB	1.270393	0.859756	-0.41064	5.44E-07
TBL3	0.846692	0.498118	-0.34857	5.44E-07
TBX2	1.322005	0.97924	-0.34276	5.44E-07
TCEB2	1.592602	1.097565	-0.49504	5.44E-07
TCF21	1.455959	1.129738	-0.32622	5.44E-07
TCIRG1	1.503268	1.046861	-0.45641	5.44E-07
TEX264	1.103587	0.745721	-0.35787	5.44E-07
TFPT	0.867548	0.516465	-0.35108	5.44E-07
TIMP1	1.727927	1.392583	-0.33534	5.44E-07
TKT	1.617794	1.292534	-0.32526	5.44E-07
TLE2	1.269178	0.922544	-0.34663	5.44E-07
TM7SF2	1.319396	0.954429	-0.36497	5.44E-07
TMA7	0.652305	0.315235	-0.33707	5.44E-07
TMED3	1.250574	0.915088	-0.33549	5.44E-07
TMED8	0.291547	0.649292	0.357745	1.21E-05
TMED9	1.576804	1.250873	-0.32593	5.44E-07
TMEM125	1.511935	1.11122	-0.40071	5.44E-07
TMEM134	1.109814	0.687343	-0.42247	5.44E-07
TMEM154	0.400006	0.732505	0.332499	1.21E-05
TMEM204	1.153057	0.804742	-0.34832	5.44E-07
TMEM205	1.406828	1.064432	-0.3424	5.44E-07
TMEM219	1.469089	1.060932	-0.40816	5.44E-07
TMEM256-	1.571267	1.107975	-0.46329	5.44E-07
TMEM258	1.942045	1.464584	-0.47746	5.44E-07
TMEM259	1.568386	1.138007	-0.43038	5.44E-07
TMEM74B	1.05827	0.671821	-0.38645	5.44E-07
TMSB10	2.572698	1.959722	-0.61298	5.44E-07
TMSB4X	2.492791	1.685914	-0.80688	5.44E-07
TMUB1	1.070708	0.645461	-0.42525	5.44E-07
TNFRSF1	1.043061	0.669	-0.37406	5.44E-07
TNIP3	0.242042	0.613624	0.371582	1.21E-05
TNK2	1.032433	0.68223	-0.3502	5.44E-07
TNNC1	1.450076	1.051813	-0.39826	5.44E-07
TOMM6	1.510223	1.006454	-0.50377	5.44E-07
TP53I13	1.092663	0.632201	-0.46046	5.44E-07
TPM2	1.517872	1.04418	-0.47369	5.44E-07
TPPP3	1.635724	1.114255	-0.52147	5.44E-07
TRABD	1.094478	0.703699	-0.39078	5.44E-07
TRADD	1.077995	0.750849	-0.32715	5.44E-07
TRAPPC2	1.228563	0.811006	-0.41756	5.44E-07
TRAPPC6	1.156101	0.695555	-0.46055	5.44E-07
TRIM28	1.361435	1.028142	-0.33329	5.44E-07
TRIM39	1.263165	0.907963	-0.3552	5.44E-07
TRIP6	1.167339	0.815336	-0.352	5.44E-07
TRMT1	0.954571	0.623145	-0.33143	5.44E-07
TRMT112	1.0157	0.676294	-0.33941	5.44E-07
TSPAN4	1.422349	0.962762	-0.45959	5.44E-07

TSPO	1.707878	1.280328	-0.42755	5.44E-07
TSSC4	0.987663	0.638087	-0.34958	5.44E-07
TST	1.100666	0.716128	-0.38454	5.44E-07
TSTD1	1.450456	0.997694	-0.45276	5.44E-07
TTBK2	0.224871	0.557917	0.333046	1.21E-05
TUBA1B	1.533068	1.177187	-0.35588	5.44E-07
TUBA1C	1.46582	1.117262	-0.34856	5.44E-07
TWF2	1.281803	0.936372	-0.34543	5.44E-07
TYMP	1.623051	1.16024	-0.46281	5.44E-07
TYROBP	2.017076	1.517129	-0.49995	5.44E-07
UBALD1	0.973912	0.615061	-0.35885	5.44E-07
UBB	2.162252	1.817078	-0.34517	5.44E-07
UBL5	1.757685	1.267727	-0.48996	5.44E-07
UBTD1	1.119015	0.768144	-0.35087	5.44E-07
UBXN1	1.428238	1.058671	-0.36957	5.44E-07
UBXN6	1.317049	0.934327	-0.38272	5.44E-07
UBXN7	0.369423	0.873036	0.503613	1.21E-05
UNC13D	1.202901	0.877425	-0.32548	5.44E-07
UNC93B1	1.178294	0.854363	-0.32393	5.44E-07
UPK3B	1.347562	1.012346	-0.33522	5.44E-07
UQCRHL	1.720798	1.345689	-0.37511	5.44E-07
URGCP	1.42958	1.002669	-0.42691	5.44E-07
UXT	1.488469	1.147274	-0.3412	5.44E-07
VAMP5	1.486354	1.027733	-0.45862	5.44E-07
VAMP8	1.833111	1.356971	-0.47614	5.44E-07
VIM	2.35121	1.961529	-0.38968	5.44E-07
VKORC1	1.427437	1.089591	-0.33785	5.44E-07
VMO1	0.863088	0.535312	-0.32778	5.44E-07
VPS28	1.548679	1.067707	-0.48097	5.44E-07
VSIG2	1.173908	0.764368	-0.40954	5.44E-07
WBP1	1.297629	0.948014	-0.34961	5.44E-07
WDR13	1.276319	0.917082	-0.35924	5.44E-07
WDR18	0.990977	0.553166	-0.43781	5.44E-07
WDR34	1.13498	0.774309	-0.36067	5.44E-07
WDR83OS	1.526158	1.08712	-0.43904	5.44E-07
WFDC2	1.597453	1.171354	-0.4261	5.44E-07
XAB2	1.100085	0.763234	-0.33685	5.44E-07
YPEL3	1.399811	1.044327	-0.35548	5.44E-07
ZAP70	0.856386	0.522426	-0.33396	5.44E-07
ZBED6	0.173182	0.615256	0.442074	1.21E-05
ZBTB16	0.681278	1.01169	0.330412	1.21E-05
ZC3HAV1L	0.313601	0.708728	0.395127	1.21E-05
ZDHHC12	0.980345	0.637852	-0.34249	5.44E-07
ZFAND2B	0.973904	0.633154	-0.34075	5.44E-07
ZGPAT	1.12566	0.715415	-0.41024	5.44E-07
ZNF121	0.483845	0.834871	0.351026	1.21E-05
ZNF205	0.6771	0.332571	-0.34453	5.44E-07
ZNF219	0.996803	0.632482	-0.36432	5.44E-07
ZNF358	1.197384	0.800242	-0.39714	5.44E-07
ZNF428	1.036167	0.693801	-0.34237	5.44E-07
ZNF444	0.991453	0.602797	-0.38866	5.44E-07
ZNF524	0.882149	0.488309	-0.39384	5.44E-07
ZNF593	1.125006	0.763674	-0.36133	5.44E-07
ZNF688	0.720799	0.350345	-0.37045	5.44E-07
ZNF791	0.622025	0.968539	0.346514	1.21E-05
ZNHIT1	1.181779	0.824538	-0.35724	5.44E-07
