# Supplementary material for: Evaluation and Interpretation of Transcriptome Data Underlying Heterogeneous Chronic Obstructive Pulmonary Disease
Source: Genomics Inform. 2019 Mar 31;17(1):e2. doi: 10.5808/GI.2019.17.1.e2 (PMC6459164; doi:10.5808/GI.2019.17.1.e2)
Supplement: Supplementary Table 2. — List of differentially expressed pairs determined by maximal information coefficient [file gi-2019-17-1-e2-suppl5.pdf]

Supplementary Table 2. List of differentially expressed pairs determined by maximal information coefficient

Gene1	Gene2	NOR	COPD	Difference
A1CF	*APOBEC1*	0.91026	0.29687	-0.61339
ABHD16A	*HM13*	0.29536	0.70433	0.40897
ACMSD	*MAGEA11*	0.6906	0.29046	-0.40014
ACTB	*ACTG1*	0.36761	0.78657	0.41896
ACTB	*ARPC1B*	0.22049	0.6484	0.42791
ACTB	*ATP5A1*	0.21327	0.64286	0.42959
ACTB	*CD81*	0.26187	0.6641	0.40223
ACTB	*CFL1*	0.25233	0.71049	0.45816
ACTB	*CSNK2B*	0.29712	0.71283	0.41571
ACTB	*DYNLL1*	0.17759	0.59702	0.41943
ACTB	*EIF3D*	0.20648	0.66509	0.45861
ACTB	*EIF3K*	0.22198	0.68719	0.46521
ACTB	*FBL*	0.17365	0.69792	0.52427
ACTB	*GNB2L1*	0.25168	0.74101	0.48933
ACTB	*HNRNPH1*	0.20252	0.60845	0.40593
ACTB	*LASP1*	0.24578	0.70179	0.45601
ACTB	*MGMT*	0.17675	0.58343	0.40668
ACTB	*MYL12B*	0.20761	0.6409	0.43329
ACTB	*OTUB1*	0.18221	0.64917	0.46696
ACTB	*PFN1*	0.24885	0.75623	0.50738
ACTB	*PPP1CA*	0.16824	0.59347	0.42523
ACTB	*RAC1*	0.23157	0.67685	0.44528
ACTB	*RPLP1*	0.2297	0.67794	0.44824
ACTB	*RPS19*	0.18255	0.67013	0.48758
ACTB	*RPS4X*	0.17226	0.61277	0.44051
ACTB	*S100A11*	0.15268	0.57572	0.42304
ACTB	*SSRP1*	0.23767	0.64258	0.40491
ACTB	*VIM*	0.1783	0.70848	0.53018
ACTG1	*BRK1*	0.21097	0.63363	0.42266
ACTG1	*CFL1*	0.20194	0.62587	0.42393
ACTG1	*LGALS13*	0.2264	0.6359	0.4095
ACTN4	*TJP1*	0.71628	0.27715	-0.43913
ACTR2	*ACTR3*	0.84491	0.38579	-0.45912
ACTR2	*ARPC5*	0.72226	0.31881	-0.40345
ADRM1	*RPN1*	0.22124	0.67247	0.45123
AFF1	*DDX6*	0.73913	0.30994	-0.42919
AHCYL1	*YAP1*	0.76229	0.34422	-0.41807
AIRE	*HIST1H3B*	0.69963	0.29844	-0.40119
AIRE	*HIST1H3C*	0.73587	0.22695	-0.50892
AIRE	*HIST1H3F*	0.70235	0.28071	-0.42164
AKT3	*APPL1*	0.76203	0.35147	-0.41056
ALDOA	*DYNLL1*	0.21945	0.66711	0.44766
ALDOA	*LGALS1*	0.22467	0.74864	0.52397
ANP32E	*FYTTD1*	0.82805	0.37141	-0.45664
ANXA11	*HLA-DRB4*	0.19461	0.63651	0.4419
AP2A1	*BIN1*	0.24925	0.65195	0.4027
APCS	*CRP*	0.78423	0.2859	-0.49833
APCS	*MBL2*	1	0.44444	-0.55556
APEX1	*LGALS1*	0.23475	0.64119	0.40644
APOA2	*APOF*	0.76891	0.36179	-0.40712
ARCN1	*COPB2*	0.8913	0.35569	-0.53561
ARCN1	*COPE*	0.73118	0.28376	-0.44742
ARF1	*COPE*	0.19114	0.74353	0.55239

https://doi.org/10.5808/GI.2019.17.1.e2

ARID1A	*ITCH*	0.67517	0.22209	-0.45308
ARR3	*FSHR*	0.7381	0.30534	-0.43276
ARR3	*OPN1MW*	0.96438	0.39877	-0.56561
ARR3	*OPN4*	0.7579	0.30211	-0.45579
ATF2	*DISC1*	0.74165	0.34137	-0.40028
ATF2	*MAPK1*	0.76697	0.30948	-0.45749
ATP4A	*GIF*	0.90386	0.3382	-0.56566
ATP5B	*P4HB*	0.26905	0.68481	0.41576
AUP1	*SPTLC1*	0.66258	0.25706	-0.40552
AVP	*AVPR1B*	1	0.4728	-0.5272
BAG6	*NR1H2*	0.25228	0.66154	0.40926
BAG6	*PRDX5*	0.24232	0.66286	0.42054
BAP1	*UBE2V1*	0.19427	0.70784	0.51357
BCL2	*PPP3R1*	0.66452	0.23996	-0.42456
BCL7C	*SMARCA4*	0.23851	0.64159	0.40308
BIN1	*DNM2*	0.22089	0.62113	0.40024
BOLL	*DAZ2*	0.93902	0.52329	-0.41573
BSG	*OS9*	0.25737	0.78148	0.52411
BSG	*RANBP3*	0.25721	0.66659	0.40938
C10orf62	*KRTAP4-1*	0.78474	0.34587	-0.43887
CALM1	*HUWE1*	0.72377	0.31166	-0.41211
CALM1	*RDX*	0.69489	0.27266	-0.42223
CAND1	*CANX*	0.77236	0.35287	-0.41949
CAND1	*CAPRIN1*	0.82692	0.3758	-0.45112
CAND1	*DDX6*	0.91016	0.49315	-0.41701
CAND1	*FUBP3*	0.66941	0.2487	-0.42071
CAND1	*H2AFV*	0.72391	0.30544	-0.41847
CAND1	*HSPA4*	0.79785	0.36416	-0.43369
CAND1	*OPA1*	0.7981	0.37892	-0.41918
CAND1	*POLR2B*	0.77548	0.37225	-0.40323
CAND1	*SERBP1*	0.64253	0.23671	-0.40582
CANX	*GANAB*	0.77189	0.35928	-0.41261
CANX	*LMAN1*	0.80841	0.40531	-0.4031
CANX	*SUMO3*	0.74426	0.31763	-0.42663
CAPN1	*EIF6*	0.29775	0.73097	0.43322
CARD17	*CARD18*	0.81464	0.31096	-0.50368
CAV1	*SDPR*	0.63497	0.20806	-0.42691
CCDC185	*KRTAP4-1*	0.78799	0.35687	-0.43112
CCT3	*TRIM28*	0.17422	0.73042	0.5562
CD2	*LCK*	0.78875	0.3226	-0.46615
CD3E	*CD3G*	0.66462	0.24861	-0.41601
CD63	*VKORC1*	0.28251	0.72004	0.43753
CD74	*HLA-DRB1*	0.17443	0.6255	0.45107
CD81	*RPL11*	0.24525	0.65262	0.40737
CD81	*RPL27*	0.21217	0.66267	0.4505
CD81	*RPS4Y1*	0.24417	0.65177	0.4076
CD81	*VKORC1*	0.25933	0.73582	0.47649
CDC5L	*WRN*	0.24803	0.70293	0.4549
CDH7	*FGF21*	0.93478	0.36169	-0.57309
CDIPT	*ILK*	0.2376	0.64687	0.40927
CGA	*CGB*	1	0.34864	-0.65136
CGA	*FSHR*	0.76816	0.24981	-0.51835
CGA	*TSHB*	0.827	0.22916	-0.59784
CHMP3	*UBE2H*	0.71173	0.30583	-0.4059
CHMP4A	*VPS4A*	0.1973	0.59782	0.40052
CLIC1	*RPLP1*	0.28034	0.76256	0.48222

CLPS	*LIPF*	0.93478	0.33108	-0.6037
CLPS	*PNLIP*	1	0.55914	-0.44086
CLPS	*PNLIPRP2*	0.97005	0.42691	-0.54314
COPB2	*LMAN1*	0.79941	0.36443	-0.43498
COPS6	*TAF15*	0.25871	0.69474	0.43603
CPN1	*CPN2*	0.70745	0.27905	-0.4284
CREB1	*DR1*	0.90375	0.44986	-0.45389
CREB1	*ETS1*	0.62616	0.22082	-0.40534
CREB3	*FIS1*	0.25532	0.69855	0.44323
CREB3	*RABAC1*	0.26777	0.68781	0.42004
CREB3	*VKORC1*	0.28365	0.70598	0.42233
CRK	*DDX6*	0.74533	0.32194	-0.42339
CRK	*IRS1*	0.65234	0.23256	-0.41978
CRP	*HIST1H1A*	0.72274	0.3074	-0.41534
CRYAA	*CRYBA1*	0.72308	0.31935	-0.40373
CRYAA	*CRYGC*	0.97826	0.49517	-0.48309
CRYAA	*CRYGD*	0.88114	0.36344	-0.5177
CRYAA	*LALBA*	0.94723	0.38997	-0.55726
CRYGC	*CRYGD*	1	0.41279	-0.58721
CSH1	*PCBP1*	0.25263	0.69009	0.43746
CSH1	*PFDN5*	0.19568	0.63399	0.43831
CSN1S1	*KLK6*	0.79385	0.26718	-0.52667
CSN2	*CSN3*	1	0.50003	-0.49997
CSNK1A1	*SYNCRIP*	0.77511	0.33148	-0.44363
CSNK2B	*PRMT1*	0.29467	0.73827	0.4436
CSNK2B	*RPS8*	0.2582	0.75469	0.49649
CSNK2B	*SSRP1*	0.25109	0.65665	0.40556
CSRP3	*LIMS3*	0.8994	0.29988	-0.59952
CSRP3	*MYF6*	0.92756	0.30091	-0.62665
CSRP3	*MYOG*	0.97791	0.29312	-0.68479
CSTF2T	*UBQLN1*	0.67741	0.24034	-0.43707
CTCFL	*HIST1H3B*	0.82443	0.30786	-0.51657
CTCFL	*HIST1H3C*	0.79626	0.29743	-0.49883
CTCFL	*HIST1H3F*	0.873	0.34061	-0.53239
CTCFL	*HIST1H3J*	0.75062	0.29356	-0.45706
CTNNA1	*DLG1*	0.88114	0.38593	-0.49521
CTNNA1	*TJP1*	0.81418	0.33712	-0.47706
CTNNA1	*VCL*	0.66939	0.25053	-0.41886
CUL3	*KRR1*	0.8582	0.4555	-0.4027
CUL3	*PHAX*	0.74383	0.33044	-0.41339
CUL3	*PRKAR1A*	0.7972	0.37489	-0.42231
CYC1	*NDUFB10*	0.25327	0.76991	0.51664
CYHR1	*VPS26A*	0.81137	0.27151	-0.53986
CYP11B1	*CYP11B2*	0.86667	0.44703	-0.41964
DAG1	*UTRN*	0.76295	0.20797	-0.55498
DAZ2	*DAZL*	0.84639	0.35052	-0.49587
DDX6	*PNRC2*	0.81942	0.34141	-0.47801
DEFA5	*PRSS1*	1	0.338	-0.662
DEK	*EP300*	0.68832	0.24041	-0.44791
DISC1	*EXOC4*	0.82033	0.35407	-0.46626
DISC1	*SMC3*	0.83401	0.38558	-0.44843
DISC1	*XRN2*	0.73708	0.31866	-0.41842
DMBX1	*OTX2*	0.71342	0.29157	-0.42185
DNAJB9	*RFK*	0.76855	0.28984	-0.47871
DNMT3L	*HIST1H3B*	0.9027	0.34927	-0.55343
DNMT3L	*HIST1H3C*	0.86813	0.26312	-0.60501

DNMT3L	*HIST1H3F*	0.94001	0.33261	-0.6074
DNMT3L	*HIST1H3J*	0.82436	0.31501	-0.50935
DNMT3L	*HIST1H4A*	0.84669	0.28707	-0.55962
DNMT3L	*HIST1H4B*	0.78765	0.24712	-0.54053
DNMT3L	*HIST1H4D*	0.88418	0.27398	-0.6102
DNMT3L	*HIST1H4F*	0.92911	0.25352	-0.67559
DNMT3L	*HIST1H4J*	1	0.25662	-0.74338
DNMT3L	*HIST1H4K*	0.80505	0.34621	-0.45884
DNMT3L	*HIST1H4L*	0.97826	0.39956	-0.5787
DYNC1LI2	*DYNLT3*	0.69085	0.23784	-0.45301
DYNLL1	*GNB2L1*	0.26465	0.74787	0.48322
EEF2	*RPS24*	0.25869	0.68654	0.42785
EEF2	*RPS4X*	0.20359	0.76721	0.56362
EEF2	*RPS6*	0.33832	0.81455	0.47623
EIF3D	*RPS4X*	0.26852	0.73703	0.46851
EIF3I	*EIF3K*	0.27875	0.69283	0.41408
EIF3I	*VIM*	0.21408	0.64994	0.43586
EIF3K	*GNB2L1*	0.23245	0.70148	0.46903
ELSPBP1	*MYF6*	0.87629	0.40134	-0.47495
ELSPBP1	*MYOG*	0.91307	0.39882	-0.51425
EP300	*H3F3B*	0.70637	0.29174	-0.41463
EP300	*NBN*	0.77082	0.31915	-0.45167
EP300	*PPP2R5C*	0.70752	0.28721	-0.42031
ERBB2IP	*SHOC2*	0.71828	0.31684	-0.40144
ESRRA	*PNRC2*	0.77386	0.34252	-0.43134
EWSR1	*PCBP1*	0.18683	0.65075	0.46392
FBL	*RPS24*	0.2721	0.75204	0.47994
FBL	*RPS4X*	0.28913	0.7088	0.41967
FBXW11	*YAP1*	0.7976	0.37137	-0.42623
G3BP2	*RIOK2*	0.85174	0.38936	-0.46238
G3BP2	*RNH1*	0.72284	0.32026	-0.40258
G3BP2	*TLN1*	0.62776	0.2052	-0.42256
GAGE1	*TERF2IP*	0.32252	0.74755	0.42503
GH1	*HIST1H2BI*	0.82924	0.23253	-0.59671
GIGYF2	*PRPF8*	0.72019	0.31463	-0.40556
GNB2L1	*RPL7A*	0.21064	0.62035	0.40971
GNB2L1	*RPS8*	0.25317	0.6638	0.41063
GNGT1	*RHO*	0.78497	0.30273	-0.48224
GPHA2	*GPHB5*	0.917	0.21123	-0.70577
GSTK1	*PCBP1*	0.19304	0.59584	0.4028
GSTK1	*UNC45A*	0.22029	0.64753	0.42724
GULP1	*ITGB1*	0.76957	0.2465	-0.52307
HERPUD1	*SYVN1*	0.21874	0.61894	0.4002
HGS	*UBQLN1*	0.76283	0.35352	-0.40931
HIST1H1A	*HIST1H3A*	0.83164	0.34426	-0.48738
HIST1H1A	*HIST1H3B*	0.94689	0.36348	-0.58341
HIST1H1A	*HIST1H3C*	0.94723	0.32829	-0.61894
HIST1H1A	*HIST1H3F*	0.97791	0.31731	-0.6606
HIST1H1A	*HIST1H3G*	0.86956	0.31764	-0.55192
HIST1H1A	*HIST1H3I*	0.84874	0.24562	-0.60312
HIST1H1A	*HIST1H3J*	0.917	0.32206	-0.59494
HIST1H2A	*HIST1H2B*	1	0.42889	-0.57111
HIST1H2A	*HIST1H2B*	1	0.41969	-0.58031
HIST1H2A	*HIST1H3A*	0.74505	0.23878	-0.50627
HIST1H2A	*HIST1H3B*	0.96438	0.2626	-0.70178
HIST1H2A	*HIST1H3C*	0.93478	0.27182	-0.66296

HIST1H2A	*HIST1H3F*	0.99965	0.29049	-0.70916
HIST1H2A	*HIST1H3G*	0.78243	0.22825	-0.55418
HIST1H2A	*HIST1H3I*	0.84355	0.23266	-0.61089
HIST1H2A	*HIST1H3J*	0.88587	0.22908	-0.65679
HIST1H2A	*HIST1H3G*	0.59607	0.18515	-0.41092
HIST1H2B	*HIST1H3A*	0.94011	0.29636	-0.64375
HIST1H2B	*HIST1H3B*	1	0.33775	-0.66225
HIST1H2B	*HIST1H3C*	1	0.3457	-0.6543
HIST1H2B	*HIST1H3F*	1	0.41161	-0.58839
HIST1H2B	*HIST1H3G*	0.9576	0.35615	-0.60145
HIST1H2B	*HIST1H3I*	1	0.32171	-0.67829
HIST1H2B	*HIST1H3J*	0.97005	0.37747	-0.59258
HIST1H3A	*HIST1H4A*	0.78726	0.23576	-0.5515
HIST1H3A	*HIST1H4B*	0.73378	0.27913	-0.45465
HIST1H3A	*HIST1H4D*	0.9042	0.28028	-0.62392
HIST1H3A	*HIST1H4F*	0.83506	0.24876	-0.5863
HIST1H3A	*HIST1H4J*	0.88842	0.27741	-0.61101
HIST1H3A	*HIST1H4K*	0.77114	0.24493	-0.52621
HIST1H3A	*HIST1H4L*	0.88114	0.31778	-0.56336
HIST1H3A	*LALBA*	0.88114	0.30882	-0.57232
HIST1H3B	*HIST1H4A*	0.99965	0.28498	-0.71467
HIST1H3B	*HIST1H4B*	0.96971	0.20983	-0.75988
HIST1H3B	*HIST1H4C*	0.64173	0.22936	-0.41237
HIST1H3B	*HIST1H4D*	0.94689	0.2661	-0.68079
HIST1H3B	*HIST1H4F*	0.99965	0.25641	-0.74324
HIST1H3B	*HIST1H4I*	0.71705	0.26291	-0.45414
HIST1H3B	*HIST1H4J*	1	0.28504	-0.71496
HIST1H3B	*HIST1H4K*	0.95726	0.32205	-0.63521
HIST1H3B	*HIST1H4L*	1	0.39688	-0.60312
HIST1H3B	*LALBA*	1	0.32839	-0.67161
HIST1H3C	*HIST1H4A*	0.93478	0.22476	-0.71002
HIST1H3C	*HIST1H4B*	0.90483	0.24286	-0.66197
HIST1H3C	*HIST1H4C*	0.63135	0.20362	-0.42773
HIST1H3C	*HIST1H4D*	1	0.23978	-0.76022
HIST1H3C	*HIST1H4E*	0.61072	0.19404	-0.41668
HIST1H3C	*HIST1H4F*	0.97005	0.27854	-0.69151
HIST1H3C	*HIST1H4I*	0.71859	0.23073	-0.48786
HIST1H3C	*HIST1H4J*	0.97826	0.27904	-0.69922
HIST1H3C	*HIST1H4K*	0.91728	0.22624	-0.69104
HIST1H3C	*HIST1H4L*	0.97826	0.31907	-0.65919
HIST1H3C	*LALBA*	0.97826	0.36001	-0.61825
HIST1H3F	*HIST1H4A*	0.96971	0.25442	-0.71529
HIST1H3F	*HIST1H4B*	0.96971	0.22676	-0.74295
HIST1H3F	*HIST1H4D*	0.96971	0.23495	-0.73476
HIST1H3F	*HIST1H4F*	0.97791	0.29723	-0.68068
HIST1H3F	*HIST1H4I*	0.84685	0.31009	-0.53676
HIST1H3F	*HIST1H4J*	0.97826	0.28537	-0.69289
HIST1H3F	*HIST1H4K*	0.99965	0.19773	-0.80192
HIST1H3F	*HIST1H4L*	0.97826	0.28916	-0.6891
HIST1H3F	*LALBA*	0.97826	0.3007	-0.67756
HIST1H3G	*HIST1H4A*	0.86956	0.32649	-0.54307
HIST1H3G	*HIST1H4B*	0.86956	0.21632	-0.65324
HIST1H3G	*HIST1H4D*	0.92945	0.26935	-0.6601
HIST1H3G	*HIST1H4F*	0.96971	0.24867	-0.72104
HIST1H3G	*HIST1H4J*	0.93432	0.21044	-0.72388
HIST1H3G	*HIST1H4K*	0.90027	0.23261	-0.66766

HIST1H3G	*HIST1H4L*	0.93432	0.41417	-0.52015
HIST1H3G	*LALBA*	0.93432	0.25527	-0.67905
HIST1H3I	*HIST1H4A*	0.85217	0.20602	-0.64615
HIST1H3I	*HIST1H4B*	0.76967	0.21938	-0.55029
HIST1H3I	*HIST1H4D*	0.87468	0.25776	-0.61692
HIST1H3I	*HIST1H4F*	0.92489	0.28923	-0.63566
HIST1H3I	*HIST1H4J*	0.97826	0.36225	-0.61601
HIST1H3I	*HIST1H4K*	0.79678	0.27365	-0.52313
HIST1H3I	*HIST1H4L*	0.94745	0.28428	-0.66317
HIST1H3I	*LALBA*	0.97826	0.37456	-0.6037
HIST1H3J	*HIST1H4A*	0.86816	0.21716	-0.651
HIST1H3J	*HIST1H4B*	0.87577	0.21596	-0.65981
HIST1H3J	*HIST1H4D*	0.85919	0.21242	-0.64677
HIST1H3J	*HIST1H4F*	0.90153	0.27309	-0.62844
HIST1H3J	*HIST1H4J*	0.94723	0.29221	-0.65502
HIST1H3J	*HIST1H4K*	0.87891	0.30628	-0.57263
HIST1H3J	*HIST1H4L*	1	0.26445	-0.73555
HIST1H3J	*LALBA*	0.94723	0.36431	-0.58292
HIST1H4A	*HIST3H3*	0.80168	0.26805	-0.53363
HIST1H4A	*PRAME*	0.70274	0.28542	-0.41732
HIST1H4B	*HIST3H3*	0.75148	0.28131	-0.47017
HIST1H4B	*PRAME*	0.67151	0.19502	-0.47649
HIST1H4D	*HIST3H3*	0.81479	0.23095	-0.58384
HIST1H4D	*PRAME*	0.68631	0.26073	-0.42558
HIST1H4F	*HIST3H3*	0.90053	0.21579	-0.68474
HIST1H4F	*PRAME*	0.7093	0.23403	-0.47527
HIST1H4J	*HIST3H3*	0.91016	0.26531	-0.64485
HIST1H4J	*PRAME*	0.76808	0.238	-0.53008
HIST1H4K	*HIST3H3*	0.85048	0.26397	-0.58651
HIST1H4K	*PRAME*	0.68276	0.2094	-0.47336
HIST1H4L	*HIST3H3*	0.90438	0.37233	-0.53205
HIST1H4L	*PRAME*	0.75423	0.33898	-0.41525
HNRNPH2	*RIOK2*	0.78124	0.38089	-0.40035
HNRNPR	*SYNCRIP*	0.7495	0.28403	-0.46547
HNRNPU	*LBR*	0.69477	0.2811	-0.41367
HNRNPU	*LSM14A*	0.76186	0.32063	-0.44123
HNRNPU	*MRGBP*	0.69415	0.27219	-0.42196
HSF1	*SMARCA4*	0.20894	0.62689	0.41795
HTRA2	*VPS4B*	0.768	0.33436	-0.43364
HUWE1	*PRKAR1A*	0.78899	0.34895	-0.44004
HUWE1	*PRPF8*	0.88022	0.47927	-0.40095
HUWE1	*SNRNP200*	0.88593	0.30974	-0.57619
IAPP	*PDX1*	0.88706	0.38974	-0.49732
IFITM3	*VKORC1*	0.26749	0.72722	0.45973
ILK	*VKORC1*	0.1906	0.71281	0.52221
ISL1	*LHX3*	0.66405	0.25558	-0.40847
ITCH	*NUMB*	0.68754	0.24604	-0.4415
ITCH	*TAX1BP1*	0.67183	0.25451	-0.41732
ITGAV	*ITGB1*	0.74216	0.25319	-0.48897
ITGB1	*RAB8B*	0.75659	0.30329	-0.4533
ITGB1	*TGOLN2*	0.72113	0.31904	-0.40209
ITGB1	*TLN1*	0.74561	0.24646	-0.49915
KIAA1033	*ROCK1*	0.82125	0.42003	-0.40122
KIAA1429	*SNRNP200*	0.7795	0.36896	-0.41054
KIR3DS1	*NDUFAF3*	0.29566	0.70159	0.40593
KMT2A	*TAF6*	0.17787	0.58393	0.40606

KRTAP3-2	*KRTAP4-1*	0.81914	0.32017	-0.49897
KRTAP4-1	*SMCP*	0.88509	0.31977	-0.56532
KRTAP4-1	*TCEB3B*	0.7301	0.31362	-0.41648
LEPR	*SNX4*	0.74112	0.33239	-0.40873
LGALS1	*LGALS3BP*	0.17383	0.6419	0.46807
LHX3	*POU4F3*	0.76006	0.26596	-0.4941
LMAN1	*TMED5*	0.81995	0.35054	-0.46941
LZTS2	*RBPMS*	0.21342	0.6541	0.44068
MAGEA11	*TEX37*	0.97791	0.47748	-0.50043
MAGEA11	*ZSWIM2*	0.7904	0.38528	-0.40512
MAP4	*TLN1*	0.76346	0.25933	-0.50413
MAPK1	*NCOA3*	0.7625	0.34679	-0.41571
MAPK1	*PPM1A*	0.81939	0.36101	-0.45838
MC4R	*NPY*	0.77433	0.23909	-0.53524
MEP1B	*NPY*	0.73828	0.24748	-0.4908
MLF2	*TXNDC12*	0.23538	0.69758	0.4622
MLN	*MLNR*	0.76692	0.28259	-0.48433
MMGT1	*TM9SF2*	0.72091	0.29347	-0.42744
MMP8	*UMOD*	0.7965	0.33225	-0.46425
MRPL32	*MRPL37*	0.1987	0.65765	0.45895
MSN	*PAFAH1B2*	0.71505	0.26601	-0.44904
MSN	*PPM1A*	0.69343	0.2833	-0.41013
MTNR1A	*MTNR1B*	0.87315	0.36284	-0.51031
MUC7	*SMR3B*	0.93478	0.39793	-0.53685
MYH6	*MYL7*	0.88953	0.36547	-0.52406
MYOG	*TRIM43*	1	0.50251	-0.49749
NBN	*XRCC5*	0.7362	0.31767	-0.41853
NDUFS1	*STX12*	0.71596	0.31457	-0.40139
NME1	*PCBP1*	0.22114	0.747	0.52586
NPY	*NPY2R*	0.74316	0.28204	-0.46112
OAZ1	*PSMB4*	0.22335	0.65784	0.43449
OBP2A	*OR1G1*	0.86865	0.37482	-0.49383
OS9	*PPIB*	0.25312	0.66895	0.41583
OS9	*RPN1*	0.25553	0.66174	0.40621
OST4	*RPN1*	0.21179	0.66715	0.45536
OTUB1	*UBE2V1*	0.21946	0.73044	0.51098
P4HB	*PRKCSH*	0.28959	0.71031	0.42072
PAFAH1B2	*YWHAG*	0.67017	0.2513	-0.41887
PCBP1	*PCBP2*	0.22509	0.64513	0.42004
PCBP1	*RBM42*	0.18626	0.61686	0.4306
PDX1	*SLC2A2*	0.84347	0.35842	-0.48505
PJA2	*PRKAR1A*	0.85027	0.43881	-0.41146
PLEKHF2	*SDPR*	0.7003	0.22673	-0.47357
PNRC2	*SPAG9*	0.62588	0.20404	-0.42184
PPIB	*VKORC1*	0.30742	0.76003	0.45261
PPM1G	*SNRNP70*	0.22137	0.71546	0.49409
PPM1G	*STAT6*	0.23849	0.64306	0.40457
PPP1CC	*RPRD2*	0.79694	0.30461	-0.49233
PPP2R1A	*STRN*	0.3183	0.72444	0.40614
PPP3CA	*UTRN*	0.72385	0.29392	-0.42993
PRKACG	*TNP1*	0.83143	0.2325	-0.59893
PRKAR1A	*PRPF40A*	0.72314	0.29871	-0.42443
PRKAR1A	*SETD7*	0.68483	0.28199	-0.40284
PRPF6	*RPS24*	0.21318	0.61792	0.40474
PSMB1	*PSMB5*	0.22114	0.64996	0.42882
PSMB1	*PSMB6*	0.24012	0.64415	0.40403

PSMB1	*PSMC5*	0.24134	0.66889	0.42755
PSMB4	*PSMD3*	0.2477	0.69506	0.44736
PSMB5	*PSMC5*	0.32978	0.74739	0.41761
PSMB6	*PSMD3*	0.21642	0.64685	0.43043
PSMB6	*PSME1*	0.20678	0.62467	0.41789
PSMC5	*PSMD3*	0.19115	0.61993	0.42878
PSMC5	*VIM*	0.17893	0.60623	0.4273
RAD21	*SMC3*	0.84078	0.3297	-0.51108
RAD23B	*UBQLN1*	0.72011	0.28968	-0.43043
RAD50	*RFC1*	0.82871	0.41114	-0.41757
RB1	*SETD7*	0.88114	0.4286	-0.45254
RBM10	*SF1*	0.30277	0.74388	0.44111
RDX	*ROCK2*	0.69209	0.25925	-0.43284
REG1A	*REG3A*	0.88309	0.27062	-0.61247
RIOK2	*VPS26A*	0.7794	0.35586	-0.42354
RNF113B	*UBE2U*	0.71542	0.30951	-0.40591
RPL10	*RPL4*	0.78381	0.33792	-0.44589
RPL10	*RPL6*	0.77421	0.35166	-0.42255
RPL11	*RPL30*	0.21118	0.61291	0.40173
RPL11	*RPL8*	0.16766	0.71283	0.54517
RPL11	*RPLP1*	0.20205	0.63353	0.43148
RPL11	*RPS24*	0.20798	0.65485	0.44687
RPL11	*RPS4X*	0.24401	0.72699	0.48298
RPL11	*UBB*	0.2044	0.72129	0.51689
RPL12	*RPS3A*	0.82948	0.37554	-0.45394
RPL18A	*RPS27A*	0.67896	0.26756	-0.4114
RPL30	*RPL31*	0.26573	0.87755	0.61182
RPL30	*RPS3*	0.28619	0.7006	0.41441
RPL31	*RPL37A*	0.25986	0.76238	0.50252
RPL6	*RPLP0*	0.88229	0.40345	-0.47884
RPL6	*RPS27A*	0.89679	0.47124	-0.42555
RPL7A	*RPS2*	0.7622	0.36161	-0.40059
RPL7A	*RPS3A*	0.79389	0.39263	-0.40126
RPL8	*RPS4X*	0.35415	0.75747	0.40332
RPLP2	*RPS24*	0.27739	0.70679	0.4294
RPS20	*RPS4X*	0.24504	0.66089	0.41585
RPS24	*RPS4X*	0.3465	0.76791	0.42141
RPS24	*RPS8*	0.44255	0.85303	0.41048
RPS4X	*RPS6*	0.16017	0.61573	0.45556
RPS4X	*RPS8*	0.34657	0.75092	0.40435
RPS4X	*SNU13*	0.23658	0.65323	0.41665
S100A16	*S100A7A*	0.19815	0.59836	0.40021
SF1	*TAF15*	0.21941	0.62795	0.40854
SF3B6	*SNRPD2*	0.17942	0.59739	0.41797
SNRPB	*SNRPD2*	0.25164	0.67163	0.41999
SNU13	*SURF4*	0.20898	0.62302	0.41404
SP3	*TGFBR2*	0.7484	0.3057	-0.4427
STAG2	*WAPL*	0.86465	0.42168	-0.44297
TBC1D5	*VPS26A*	0.80298	0.34136	-0.46162
TRIM43	*TRIM8*	0.20044	0.68126	0.48082
UBE2U	*ZSWIM2*	0.82545	0.37885	-0.4466
VPS26A	*VPS4B*	0.85007	0.38545	-0.46462
YWHAG	*YWHAQ*	0.7589	0.33125	-0.42765

NOR, normal; COPD, chronic obstructive pulmonary disease.
